# Risk Factors and Outcomes of Premature Rupture of Membranes Among Women in the Middle East and North Africa: Mapping Review

**DOI:** 10.3390/jcm15103938

**Published:** 2026-05-20

**Authors:** Anna Nimer, Darya Smetanina, Shamsa Al Awar, Nusrat Ferdouse, Anne-Sophie Le Floch, Reem Bolbol, Yauhen Statsenko, Renata Jaczynska, Marwa Alhaj Ahmad, Luai A. Ahmed, Kornelia Zaręba

**Affiliations:** 1 Department of Obstetrics and Gynaecology, College of Medicine and Health Sciences, United Arab Emirates University, Al Ain 15551, United Arab Emirates; daryasm@uaeu.ac.ae (D.S.); sawar@uaeu.ac.ae (S.A.A.); 700045788@uaeu.ac.ae (N.F.); kzareba@uaeu.ac.ae (K.Z.); 2 Institute of Public Health, College of Medicine and Health Sciences, United Arab Emirates University, Al Ain 15551, United Arab Emirates; 700049263@uaeu.ac.ae (A.-S.L.F.); marwa.alhaj@uaeu.ac.ae (M.A.A.); luai.ahmed@uaeu.ac.ae (L.A.A.); 3 Department of Obstetrics and Gynaecology, Tawam Hospital, Al Ain 15258, United Arab Emirates; reemaabolbol@gmail.com; 4 Department of Radiology, College of Medicine and Health Sciences, United Arab Emirates University, Al Ain 15551, United Arab Emirates; e.a.statsenko@uaeu.ac.ae; 5 Department of Obstetrics, Perinatology and Gynaecology, Medical University of Warsaw, 02-091 Warsaw, Poland; renatajaczynska@o2.pl; 6 Zayed Centre for Health Sciences, United Arab Emirates University, Al Ain 15551, United Arab Emirates

**Keywords:** PROM, PPROM, pregnancy complications, Middle East, prematurity, mapping review

## Abstract

**Background/Objectives**: Term and preterm premature ruptures of membranes (PROM and PPROM) are serious pregnancy complications associated with adverse maternal and neonatal outcomes. Although widely studied in the global literature, data on the risk factors and outcomes of PROM and PPROM in the Middle East and North Africa (MENA) region remain limited. This mapping review aimed to identify and assess existing evidence and highlight gaps in knowledge regarding risk factors for PROM, including preterm PROM, and related maternal and neonatal outcomes among women in the region. **Methods**: We conducted a comprehensive and systematic search of articles published in English and Arabic between January 2000 and June 2025 across Scopus, Embase, Web of Science, and PubMed/Medline. Eligible studies included observational and interventional studies conducted in MENA countries. Data were extracted and synthesised using thematic mapping. **Results**: Out of 5359 retrieved records, 136 met the inclusion criteria. The main study design was cross-sectional (51 studies), followed by case–control (41), cohort (26), and 15 randomised controlled trials. The geographic distribution of the evidence varied significantly. Research has mainly focused on PROM and its biological risk factors, such as infections and chronic medical conditions. Psychological and environmental factors have been the least reported. Neonatal and gestational outcomes have frequently been addressed, whereas maternal outcomes have received less attention. **Conclusions**: The findings reveal significant geographic, thematic, and methodological disparities in research throughout the MENA region. The results underscore the need for further studies on the prevention and identification of women at higher risk of PROM.

## 1. Introduction

Premature rupture of membranes (PROM) refers to the spontaneous leakage of amniotic fluid before labour begins, resulting from the loss of integrity of the amniotic sac [1]. PROM occurs from 37 weeks of gestation onwards, with a prevalence of 10–20% [2]. When this rupture occurs before 37 weeks, it is classified as preterm PROM (PPROM), which affects 1.4–13.7% of pregnancies worldwide [3].

Its prevalence in the Middle East and North Africa (MENA) region varies considerably. For instance, in Saudi Arabia, rates of 20.1% for PROM and 2.3% for PPROM were observed during a two-year study [4]. A single-centre study in Egypt reported a five-year prevalence of PPROM of 4.1% [5]. Such disparities are likely influenced by differences in healthcare systems, resource availability, and reporting biases.

The clinical significance of PROM and PPROM lies in their strong associations with adverse neonatal outcomes. Globally, PROM accounts for 18–20% of perinatal deaths [6]. Mortality rates are inversely correlated with the gestational age at the time of membrane rupture. PPROM is often associated with earlier delivery, and subsequent prematurity substantially increases the risk of neonatal mortality [7,8]. Neonatal survival also depends on other factors, including maternal health, occurrence of neonatal infections, and access to high-quality obstetric and neonatal care [9].

Besides mortality, neonates born following PROM are at increased risk of serious nonlethal complications [10], including low birth weight, early-onset neonatal sepsis, respiratory distress syndrome, retinopathy of prematurity, neurodevelopmental impairment, asphyxia, and neonatal pneumothorax [11,12,13,14,15]. Certain severe complications, mainly associated with early PPROM, such as pulmonary hypoplasia [16], can occur in term PROM for reasons that are not yet completely understood [17]. The variety of these complications reflects different biological mechanisms that intensify the critical effects of PROM and PPROM on neonatal health.

Premature membrane rupture poses a significant threat of severe maternal morbidity and mortality [14,18,19,20]. Term and preterm PROM are associated with infections, sepsis, and chorioamnionitis [14,18], which may be fatal in severe cases. National enquiries into maternal deaths have reported that previable PPROM was responsible for 0.6% of all maternal deaths in France between 2001 and 2015 [19]. In the UK, four maternal deaths occurred after preterm PROM due to chorioamnionitis in 2006–2008 [20]. PPROM and PROM are also indirectly associated with complications such as haemorrhage, postpartum haemorrhage, and anaemia. These issues arise because of prolonged exposure to oxytocin and the requirement for instrumental delivery in complicated cases.

Spontaneous membrane rupture can occur for various reasons [2,3,21]. Although genital tract infections are considered the primary cause of PROM and PPROM, other non-infectious factors can also increase the risk of these conditions [22]. A prospective cohort study reported a higher risk of PPROM among pregnant women with a body mass index (BMI) below 18 kg/m^2^, gestational diabetes, a history of PPROM, standing while working, and insulin use [22]. In another case–control study, women were more likely to encounter PROM if they had hypertension during their current pregnancy, a history of abortion, a history of caesarean section, and PROM [23].

Considering the multifactorial nature of PROM, prevention and prediction remain challenging. Multiple trials have been conducted to identify interventions that can reduce the risk of PROM and PPROM. These interventions included supplementing the diet with docosahexaenoic acid (DHA), aspirin, vitamin C alone and with vitamin E, folic acid (alone, with iron, with iron and zinc, within a multiple micronutrient supplement), zinc, calcium, and copper. However, a systematic review did not confirm a protective effect of these supplements against PPROM [24].

Although spontaneous membrane rupture has been extensively studied globally, the distribution and applicability of the existing evidence across different geopolitical contexts remain uneven. Maternal and child health remains a significant concern in the MENA region [25]. Women in the Middle East form a distinct maternal cohort characterised by population-specific demographic and clinical risk factors for pregnancy-related complications. For example, 9% of pregnancies occur during adolescence [26]. Although fertility rates in Arab countries declined over the past decade, they remain high, ranging from 1.5 births per woman in the United Arab Emirates (UAE) to 6.3 in Somalia [27].

In addition to the abovementioned demographic features, women in the Middle East experience a substantial burden of metabolic and genetic risk factors. Women of reproductive age face increasing rates of overnutrition and suffer from overweight and obesity [28]. Consequently, maternal overweight is associated with a higher likelihood of caesarean section compared to mothers of normal weight [29]. Gestational diabetes mellitus is also highly prevalent in the Middle East, reaching 20.7% in Qatar [30]. Furthermore, consanguineous marriages remain common in parts of the region and contribute to the regional burden of inherited conditions [31]. Although their direct association with PROM has not been established, a higher predisposition to genetic conditions is an important feature of the demographic and clinical context of maternal health in the region.

In the MENA region, maternal and neonatal outcomes are shaped by unique demographic, socioeconomic, and healthcare factors, including traditional practices, social norms, and healthcare deterioration in some countries [32,33]. These circumstances complicate the management of pregnancies and conditions such as PROM in the region because it is unclear to what extent the established risk factors and outcomes apply to this population. This uncertainty highlights the need for a systematic review of evidence gaps to identify and characterise the available evidence on PROM and PPROM risk factors and outcomes, specifically within the MENA region.

## 2. Materials and Methods

This mapping review systematically identified, categorised, and visually mapped the distribution of existing evidence and research gaps regarding the risk factors for PROM, including preterm PROM, and related maternal and neonatal outcomes among women in the MENA region.

### 2.1. Study Design

To answer this research question, we followed the Evidence Gap Map (EGM) methodology, which incorporates systematic literature search, screening, and coding procedures [34]. Using this approach, we visually summarised and presented the existing evidence on the risk factors and outcomes of PROM and PPROM among women in the MENA region. Literature screening was conducted in accordance with the Preferred Reporting Items for Systematic Reviews and Meta-Analyses (PRISMA) guidelines. The PRISMA checklist is available online as Supplementary Table S1 in the Supplementary File. The study protocol was registered on the Open Science Framework.

### 2.2. Data Source

One author (D.S.) conducted a systematic literature search in four biomedical databases: Scopus, Embase, the Web of Science, and PubMed/Medline. The search was performed in June 2025 and included original, peer-reviewed studies published in English and Arabic between January 2000 and June 2025. The search was limited to studies conducted in the MENA region. The search strings are available online in Supplementary Table S2.

### 2.3. Eligibility Criteria

This review analysed pregnant women aged 18 years or older and examined PROM or PPROM as either an exposure or an outcome, along with associated maternal or neonatal outcomes. The inclusion criteria for studies were as follows: (1) compared health and socioeconomic profiles of women with and without rupture of membranes; (2) compared the health status of neonates born to women with and without PROM; (3) evaluated diagnostic approaches for detecting PROM; (4) investigated maternal and neonatal outcomes of PROM or PPROM. The publications were eligible for the analysis if they were conducted in the MENA countries.

We excluded reviews, conference abstracts, theses, book chapters, editorials, and grey literature from our analysis. Studies that analysed Arab populations living outside the MENA region were also excluded from the review. Studies on pregnant women aged under 18 years were excluded from the analysis.

### 2.4. Selection Process

**Figure 1:**
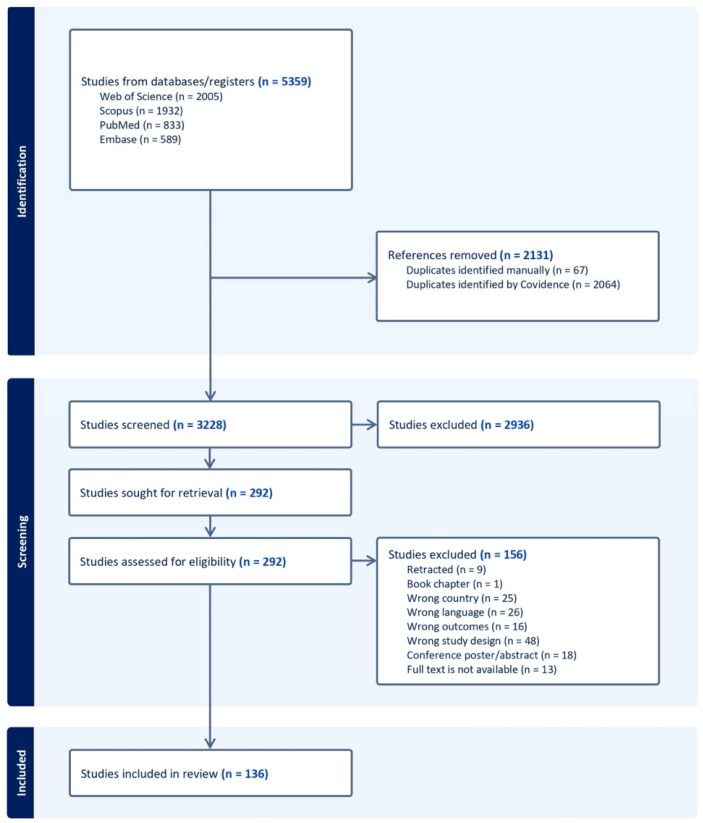
PRISMA flowchart depicting screening and selection process.

Records identified in the biomedical databases were uploaded to the web-based application for systematic reviews Covidence (Veritas Health Innovation, 2021; https://www.covidence.org/, accessed on 17 November 2025). The application was used for automatic removal of duplicates, blinded screening, and data extraction. Two reviewers (A.N. and D.S.) independently reviewed the titles and abstracts according to the inclusion and exclusion criteria. In the case of any disagreement, the reviewers reached a final decision regarding article inclusion by consensus. The same process was used to evaluate the full texts of the publications. The results of the literature screening were automatically generated and displayed with a PRISMA flowchart (Figure 1).

### 2.5. Data Collection Process

Covidence was utilised to develop a data extraction template. The extracted information included general study characteristics (first author’s name, year of publication, and country), details of the statistical approach (study design and methods for data analysis), cohort characteristics (sample size, maternal age, and gestational age at PROM), and results (type of spontaneous rupture of membranes, determinants of PROM, neonatal outcomes, and maternal outcomes).

The reviewers (A.N., A.-S.L.F., D.S., N.F., and R.B.) independently extracted data into a predetermined template. Once the two reviewers extracted information from the same article, it was marked as complete. If any discrepancies were observed, they were resolved by A.N. and D.S. After completing the data collection, the final report was downloaded from Covidence in Excel format for further analysis.

### 2.6. Quality Assessment of Individual Studies

The quality of each included study was assessed using the Joanna Briggs Institute critical appraisal tools [35]. We selected tools to evaluating analytical cross-sectional studies, cohort studies, and randomised controlled trials. The appraisal was conducted at the item level, and no overall numerical score or summary risk-of-bias category was assigned to individual studies. Three reviewers (A.-S.L.F., D.S., and N.F.) independently answered the checklist questions. The principal investigator (A.N.) and another reviewer (D.S.) verified these responses. Any disagreements were resolved through discussion with the reviewer who initially appraised the article. Details of the quality assessment are available in Supplementary Tables S4–S6.

### 2.7. Data Coding

Following data extraction and quality appraisal, the reported determinants and outcomes of PROM and PPROM were categorised into broader thematic groups. The terms retrieved from the included studies were reviewed by an obstetrician (A.N.) and a second reviewer (D.S.). Closely related terms were manually combined into a wide range of clinical themes (Supplementary Table S7). The third reviewer (K.Z.), a clinician, was consulted in cases of disagreement over assigning a specific thematic code. Maternal and neonatal infections were coded separately from maternal and neonatal outcomes because infectious complications are generally a more significant issue in PROM. This approach prevents the artificial inflation of overall outcome frequencies and enables a more precise representation of the themes on which the publications focused.

### 2.8. Data Synthesis

All analyses were performed in R (version 4.4.1; R Core Team 2025) using RStudio. The tidyverse suite was utilised for data cleaning, analysis, and visualisation. Geographic data were handled using the sf, countrycode, and rnaturalearth packages. To identify and categorise the reported risk factors for PROM and PPROM, an EGM was created to visualise the distribution of the determinant themes by PROM type. For visual mapping, rupture type was coded according to the terminology used in the original publication. This map provided a thematic overview of the most frequently investigated risk factors in relation to term and preterm membrane ruptures, revealing research focus and underexplored areas. The same mapping framework was used to synthesise data on maternal complications and neonatal outcomes after membrane rupture. A complementary EGM showed the link between risk factors and PROM outcomes.

Across all EGMs, the data reflected the number of reported relationships between study variables (such as country, PROM type, determinant, or outcome theme) rather than the number of unique studies. As individual studies can examine multiple themes or PROM categories, they contribute to several cells within the maps.

## 3. Results

### 3.1. Characteristics of the Included Studies

The search retrieved 5359 publications from four databases. After automatic deduplication, 3228 papers remained for title and abstract screening. The full texts of 292 articles were assessed. Following evaluation of the eligibility criteria, 136 publications were included in the review. The reasons for exclusion were documented (Figure 1).

**Figure 2:**
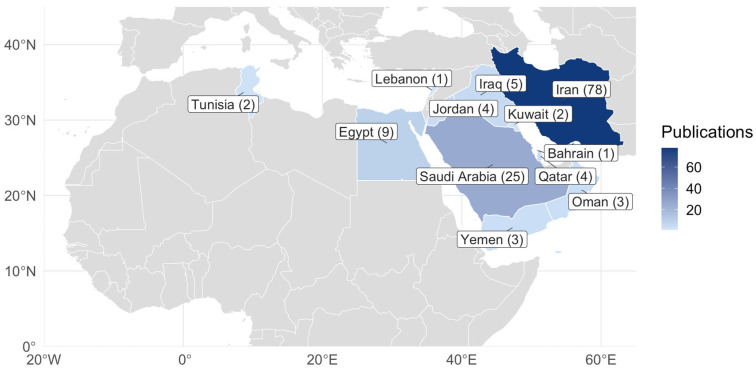
Distribution of studies on premature rupture of membranes (PROM) across the Middle East and North Africa Region.

**Figure 3:**
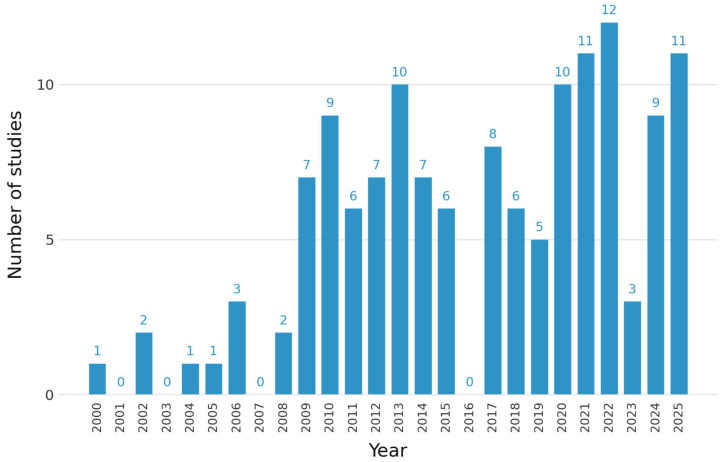
Distribution of studies on rupture of membranes (PROM and PPROM) across the Middle East and North Africa Region.

Out of 136 publications, most studies were conducted in Iran (78). Among Gulf countries, the majority of cases occurred in Saudi Arabia (25), followed by Qatar (4). No studies were conducted in the UAE. In North Africa, nine studies were published in Egypt and two in Tunisia (Figure 2). This uneven distribution of studies highlights the need for more comprehensive regional research to address the gaps in the prevention and management of PROM across the MENA region. Interest in PROM research increased in the 2010s (Figure 3).

The sample size ranged from 20 to 9773 (see Supplementary Table S3). The most common study design was cross-sectional (51 publications), followed by case–control (41) and cohort (26). Fifteen randomised controlled trials were conducted; two studies assessed the diagnostic accuracy for detecting PROM, and one study reported the overall prevalence of PROM in the studied population.

### 3.2. Quality of Included Studies

Critical appraisal of the included studies revealed variable methodological quality among the publications (see Supplementary Tables S4–S6). We identified several methodological limitations. A recurring weakness was incomplete reporting of sample size justification in several studies. The statistical analyses were limited to unadjusted group comparisons, such as Chi-square tests, exact tests, *t*-tests, or ANOVA. Although these methods can identify differences between groups, they do not establish independent associations or account for potential confounding factors. The analysis remained predominantly descriptive, and it was challenging to draw robust conclusions regarding the independent contributions of specific risk factors to PROM and its outcomes. A substantial proportion of the included literature demonstrated limited precision in classifying rupture type. Specifically, 69 of the 136 included publications reported rupture type ambiguously or used PROM definition despite including both terms and preterm pregnancies without a clear distinction between PROM and PPROM. The results suggest that the evidence is heterogeneous in methodological quality and should be interpreted with caution.

### 3.3. Distribution of Studies by Country and Research Focus

**Figure 4:**
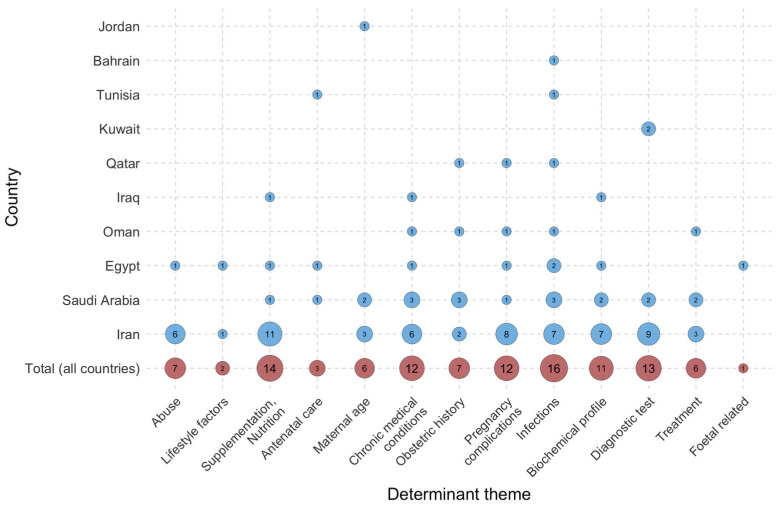
Number of studies examining risk factors for rupture of membranes (PROM and PPROM) in a specific country in the Middle East and North Africa. Circle size and numbers indicate the frequency of each theme in publications.

**Figure 5:**
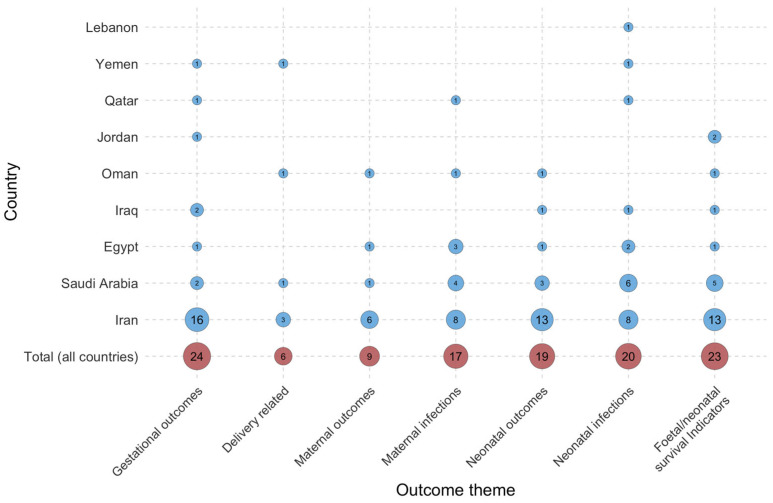
Number of studies reporting outcomes of rupture of membranes (PROM and PPROM) in a specific country in the Middle East and North Africa. Circle size and numbers indicate the frequency of each theme in publications.

Evidence of the risk factors for PROM and related outcomes is unevenly distributed across countries in the MENA region (see Figure 4 and Figure 5). Infections, particularly Group B Streptococcus (GBS), are the most studied risk factors for membrane rupture. Studies addressing infection risk factors have been conducted in seven MENA countries, indicating moderate regional coverage of this determinant [12,36,37,38,39,40,41,42,43,44,45,46,47,48,49,50]. Supplementation and nutrition were the second most common topics in the included studies [45,51,52,53,54,55,56,57,58,59,60,61,62,63]. In addition to clinical and biological factors, only a few studies have examined the sociodemographic determinants of PROM, such as abuse [64,65,66,67,68,69], maternal age [4,70,71,72,73,74], and lifestyle-related factors [45,75]. The included papers had limited information on the sociodemographic determinants of PROM. The limited availability of these data may result in omitting these factors from risk stratification and obstructing comprehensive approaches for the prevention and management of PROM.

The analysis of pregnancy-related complications revealed that gestational outcomes were the most thoroughly examined category, appearing in 24 publications (Figure 5) [4,13,14,41,59,76,77,78,79,80,81,82,83,84,85,86,87,88,89,90,91,92,93,94]. Foetal/neonatal survival indicators were documented in 23 studies conducted across six countries. Nine publications reported maternal outcomes, such as haemorrhage, oligohydramnios, manual removal of the placenta, and placental abruption. Delivery-related outcomes, including the mode of delivery, were the least studied group. The review indicated that the current evidence disproportionately emphasises neonatal and gestational outcomes, whereas maternal outcomes following PROM remain underrepresented.

Apart from determinants and outcomes, studies have focused on disease course and management (see Supplementary Figure S1). Latency was discussed in 11 publications across four countries, while management, such as antibiotic use, was explored in 7 articles.

### 3.4. Studied Risk Factors and Outcomes by PROM Type

**Figure 6:**
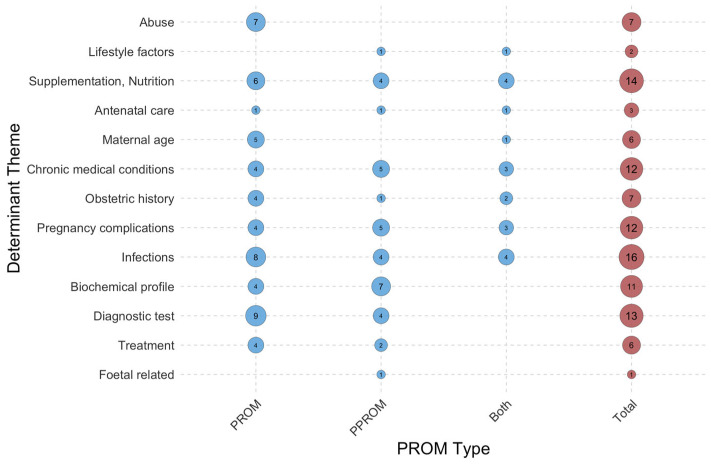
Distribution of studied risk factors of PROM and PPROM in the MENA countries. Circle size and numbers indicate the frequency of each theme in publications.

**Figure 7:**
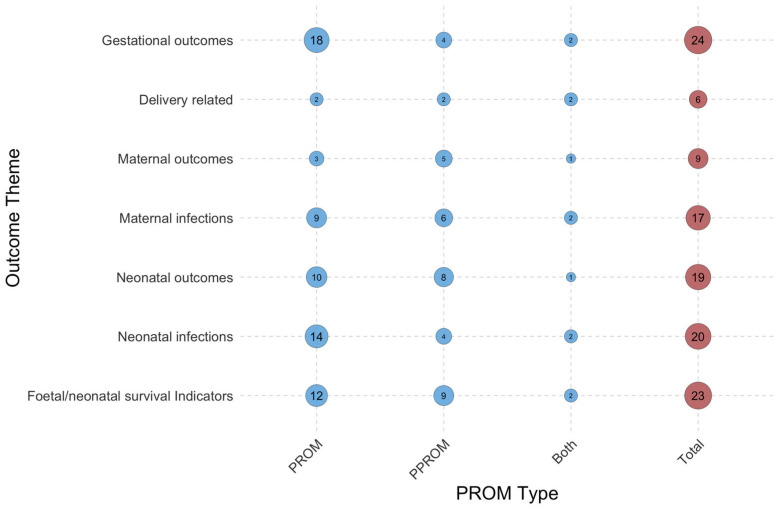
Distribution of studied outcomes of PROM and PPROM in the MENA countries. Circle size and numbers indicate the frequency of each theme in publications.

The review also examined how studies addressed the risk factors for membrane rupture before 37 weeks of gestation and at term (Figure 6 and Figure 7). Research has tended to focus on the term PROM rather than PPROM. Few studies have explored both types of membrane ruptures. While investigating the determinants of PROM, publications have mainly concentrated on the clinical and biomedical domains, including infections, pregnancy complications, diagnostic testing, biochemical profiles, and nutritional factors. Sociodemographic, behavioural, and health system–related factors (such as lifestyle, abuse, and antenatal care) have rarely been investigated, especially in studies focusing solely on PPROM or publications analysing PROM and PPROM within the same paper.

The evidence on outcomes was heavily weighted towards gestational outcomes, foetal/neonatal survival indicators, and neonatal infections. In studies focusing on mothers, maternal infections were studied more frequently than maternal and delivery-related outcomes.

The study findings revealed a significant lack of evidence regarding the risk factors and outcomes related to PPROM in research on spontaneous membrane rupture. Additionally, the studies mainly concentrated on biomedical and clinical risk factors and PROM outcomes, with sociodemographic factors seldom examined and limited to maternal age and the effects of abuse on pregnancy outcomes. This imbalance restricts a comprehensive approach to maternal and neonatal care, as well as the identification of women who are at a higher risk of PROM.

## 4. Discussion

This review revealed variability in the presentation of PROM data across studies conducted in the MENA region, both over time and between countries. Research output increased from 2009 onwards, reaching a peak in 2022. The literature has mainly focused on PROM as a risk factor for neonatal complications, including those related to infections. Most studies have focused on the term PROM. Few studies have examined the environmental and psychological risk factors of PROM and PPROM. These findings highlight the gaps and opportunities for future research on PROM determinants and outcomes in the MENA region.

### 4.1. Structural and Contextual Barriers to PROM Research in the MENA Region

PROM is a condition that connects preexisting environmental or material health risks with obstetric and neonatal outcomes, such as preterm birth. Research on PROM is often part of broader efforts to find ways to prevent neonatal mortality and morbidity rather than a unique condition that requires special attention. Although international clinical guidelines clearly define the diagnosis and management of PROM, ongoing research is vital to prevent maternal complications and neonatal death [15,95]. In particular, in the MENA region, there is a need for further insight into the causes of spontaneous membrane rupture and its complications, considering the region’s specific climatic and socioeconomic conditions, limited access to antenatal and emergency obstetric services, high medical care costs, and insurance coverage issues. Owing to complex geopolitical factors, low-income countries face challenges in reducing infant and under-five mortality rates [25,96]. Consequently, exploring PROM-specific evidence is closely linked to broader national research capacities and funding priorities.

Most countries in the MENA region face barriers in conducting research, although reducing maternal and neonatal mortality remains a higher priority [97]. Complications in studying maternal health stem from broader constraints in the healthcare sector across MENA countries. Apart from the Gulf countries, most MENA nations allocate between 2.5% and 23.09% of their Gross Domestic Product to healthcare [98]. In conflict or postwar zones, access to healthcare is limited and curative rather than preventive [99]. Furthermore, healthcare facilities lack efficient information systems to facilitate access to electronic patient profiles [100]. Owing to these barriers in healthcare systems, research efforts are limited, and topics are narrow.

The underrepresentation of environmental and psychosocial risk factors for PROM in this review may reflect broader research priorities in regions where mental health and interpersonal violence receive less attention than biological risk factors [101]. Investigating the role of such factors requires the active participation of the study subjects in clinical research, as most of these data cannot be obtained from hospital patient records because the information is not routinely collected [102]. Women may be hesitant to disclose experiences of domestic or intimate partner violence to research personnel or healthcare providers because of concerns about stigma, social repercussions, and potential harm if the disclosure becomes known to their partner [103,104]. Collectively, these barriers impede research on the influence of environmental and psychosocial factors on pregnancy outcomes and complications such as PROM.

### 4.2. Methodological Characteristics and Limitations of the Included Studies

A critical appraisal of the included studies revealed methodological weaknesses in research on membrane rupture. These limitations were reflected in the results of the quality appraisal and included incomplete reporting of sample sizes justification, limited adjustment for confounding, and misclassification of rupture type. In several studies, the condition was referred to as PROM, when preterm and term pregnancies were included in the analysis [38,42,65,80]. Considering the differences in management and outcomes, combining the two scenarios is inappropriate. Merging PROM and PPROM into a single study sample introduces misclassification bias and limits the interpretability and comparability of the findings. Another limitation of the publications was the use of basic descriptive analysis and inferential statistics (e.g., frequencies and *p*-values). However, this approach does not allow for a thorough investigation of the risks and outcomes of PROM.

These limitations illustrate the wider methodological challenges in the design and implementation of biomedical research across the region. A recent qualitative study emphasised that researchers at academic institutions in the MENA region face challenges, such as restricted access to subscription-based peer-reviewed literature, limited statistical resources, and a lack of mentorship in project management [104]. Another study identified the key barriers to conducting studies by clinicians in a hospital setting. These criteria are similar to those used in academic settings. Additionally, respondents highlighted the lack of time, complex procedures for ethical approval, difficulties in recruiting study participants, and challenges in adapting to international guidelines [105].

### 4.3. Implications of the Findings for Future Research

The EGM helped identify the most explored aspects of PROM and areas that are not thoroughly presented in the regional literature. When studying the risk factors and outcomes of PROM, the literature is heavily weighted towards the biological and clinical aspects. Infections, nutritional status, diagnostic markers, and pregnancy complications constitute most of the evidence on risk factors. These findings show that PROM is commonly studied as a condition driven by biological and clinical factors and that socioeconomic and environmental factors have received limited attention. Regarding outcomes, the focus shifted to infant-related themes, highlighting the clinical priorities for neonatal survival and health. Moreover, the studies included in this review concentrated on the immediate outcomes of PROM and did not investigate its long-term consequences. However, earlier studies from other regions have established a link between prematurity due to PPROM and moderate-to-severe neurodevelopmental impairments in children [106,107].

The literature confirms that the treatment and prevention of infections remain central to clinical care [36,108]. A curative rather than a preventive approach complicates the stratification of women by risk and hampers the development of a comprehensive preventative strategy [24]. Prophylaxis of PROM is especially challenging in low- and middle-income settings, where healthcare and research resources are limited. The findings of this review also emphasise that accumulating knowledge about preventive care and non-biological risk factors for PROM could help to develop new guidelines for antenatal care.

Further studies are required to better understand the pathophysiology and management of PPROM. The current EGM highlights a disproportionate focus on term and preterm PROM. As the prevalence of PPROM is lower than that of term PROM and it occurs in up to 3% of pregnancies, more time is needed to accumulate sufficient data for statistically sound analysis. Despite its low incidence, managing PPROM is challenging because it is associated with severe neonatal outcomes [109]. The evidence suggests the need for coordinated multicentre research efforts to accumulate PPROM cases and consistent documentation of the condition in patient files and study methodologies. This approach will improve the quality of studies and, subsequently, the risk assessment, management, and prediction of outcomes in both PROM and PPROM cases.

## 5. Strengths and Limitations

This review has the following strengths.This study followed the EGM methodology, which facilitates visual data synthesis and is easier to interpret than narrative or tabular approaches. Using EGM, the review simultaneously analyses evidence across country-specific, temporal, and thematic dimensions. Another key strength of this study is the use of broad inclusion criteria, allowing the capture of studies in which PROM was examined as a covariate, secondary outcome, or part of the cohort characteristics. This approach ensured that the review covered the full spectrum of available evidence on PROM within the MENA region. Finally, this review synthesises the evidence within a specific geopolitical region. This study provides a contextual interpretation of these existing gaps. This is particularly important when setting research priorities.

However, the review also has limitations that should be considered when interpreting its findings. The search strategy was restricted to publications in English or Arabic. Consequently, studies published in other official languages (e.g., Farsi and French) were excluded. This language restriction may have introduced the language bias in relation to the geographic and thematic distribution of studies. The EGM included studies based on relevance rather than on methodological quality. Therefore, the review offers a comprehensive overview of research topics but does not reflect the reliability of the existing evidence. Additionally, EGM does not allow for the assessment of the magnitude of associations between risk factors, the incidence of PROM, and its outcomes.

## 6. Conclusions

This study provides a thorough overview of the research gaps regarding the risk factors and outcomes of PROM and PPROM in the MENA region. These findings highlight the uneven distribution of evidence across countries and research areas. This disproportional distribution of studies corresponds to the overall trends in research outputs across the region.

A thematic gap has been identified in the disproportionate research focusing on term and preterm PROM. These studies tended to focus on term pregnancies, probably because the data were more readily available. However, more attention is required for PPROM because it is associated with more adverse outcomes in both mothers and neonates. Moreover, the studies were weighted towards the biological risk factors for spontaneous membrane rupture. Environmental and psychosocial determinants remain underexplored in studies of PROM and PPROM. These gaps are also compounded by methodological challenges in the analysed literature, such as conceptual mistakes and weak statistical approaches.

PROM management follows international guidelines that emphasise curative rather than preventive care. The MENA region is diverse in its socio-political context, and women in the region may face certain risks for PROM that have not been reported globally, such as stress from war conflicts, violence, or unique genetic disorders. Future research should prioritise multicentre studies on PPROM, improve the classification of rupture of membranes in study design and reporting, and focus on identifying modifiable biological, environmental, and psychological risk factors. These measures may help develop preventive strategies in antenatal care.

## Data Availability

No new data were created or analysed in this study.

## Supplementary Materials

The following supporting information can be downloaded at: https://www.mdpi.com/article/10.3390/jcm15103938/s1, Supplementary Table S1: PRISMA checklist is presented in Supplementary Table S2: Search strategies for biomedical databases; Supplementary Table S3: Characteristics of included studies; Supplementary Table S4: Critical Appraisal of cross-sectional studies; Supplementary Table S5: Critical appraisal of case–control studies; Supplementary Table S6: Critical appraisal of randomised-control trials; Supplementary Table S7: Thematic coding of determinants and outcomes; Supplementary Figure S1: Number of states on disease course management of PROM in a specific country in the Middle East and Africa.

## Abbreviations

The following abbreviations are used in this manuscript:

EGM	Evidence gap map
MENA	Middle East and North Africa
PPROM	Preterm premature rupture of membranes
PROM	Premature rupture of membranes
