# Supplementary material for: Risk Factors and Outcomes of Premature Rupture of Membranes Among Women in the Middle East and North Africa: Mapping Review
Source: J Clin Med. 2026 May 20;15(10):3938. doi: 10.3390/jcm15103938 (PMC13206857; doi:10.3390/jcm15103938)
Supplement: Supplementary file 1 [file jcm-15-03938-s001.zip › jcm-4285584-supplementary.pdf]

Supplementary Table S1. PRISMA checklist

Section and Topic	Item #	Checklist item	Location where item is reported
**TITLE**			
Title	1	Identify the report as a systematic review.	1
**ABSTRACT**			
Abstract	2	See the PRISMA 2020 for Abstracts checklist.	1
**INTRODUCTION**			
Rationale	3	Describe the rationale for the review in the context of existing knowledge.	2-3
Objectives	4	Provide an explicit statement of the objective(s) or question(s) the review addresses.	3
**METHODS**			
Eligibility criteria	5	Specify the inclusion and exclusion criteria for the review and how studies were grouped for the syntheses.	4
Information sources	6	Specify all databases, registers, websites, organisations, reference lists and other sources searched or consulted to identify studies. Specify the date when each source was last searched or consulted.	4
Search strategy	7	Present the full search strategies for all databases, registers and websites, including any filters and limits used.	Suppl table S2
Selection process	8	Specify the methods used to decide whether a study met the inclusion criteria of the review, including how many reviewers screened each record and each report retrieved, whether they worked independently, and if applicable, details of automation tools used in the process.	4
Data collection process	9	Specify the methods used to collect data from reports, including how many reviewers collected data from each report, whether they worked independently, any processes for obtaining or confirming data from study investigators, and if applicable, details of automation tools used in the process.	4-5
Data items	10a	List and define all outcomes for which data were sought. Specify whether all results that were compatible with each outcome domain in each study were sought (e.g. for all measures, time points, analyses), and if not, the methods used to decide which results to collect.	5
	10b	List and define all other variables for which data were sought (e.g. participant and intervention characteristics, funding sources). Describe any assumptions made about any missing or unclear information.	5
Study risk of bias assessment	11	Specify the methods used to assess risk of bias in the included studies, including details of the tool(s) used, how many reviewers assessed each study and whether they worked independently, and if applicable, details of automation tools used in the process.	NA
Effect measures	12	Specify for each outcome the effect measure(s) (e.g. risk ratio, mean difference) used in the synthesis or presentation of results.	NA
Synthesis methods	13a	Describe the processes used to decide which studies were eligible for each synthesis (e.g. tabulating the study intervention characteristics and comparing against the planned groups for each synthesis (item #5)).	5
	13b	Describe any methods required to prepare the data for presentation or synthesis, such as handling of missing summary statistics, or data conversions.	5
	13c	Describe any methods used to tabulate or visually display results of individual studies and syntheses.	5
	13d	Describe any methods used to synthesize results and provide a rationale for the choice(s). If meta-analysis was performed, describe the model(s), method(s) to identify the presence and extent of statistical heterogeneity, and software package(s) used.	5
	13e	Describe any methods used to explore possible causes of heterogeneity among study results (e.g. subgroup analysis, meta-regression).	NA
	13f	Describe any sensitivity analyses conducted to assess robustness of the synthesized results.	NA

Section and Topic	Item #	Checklist item	Location where item is reported
Reporting bias assessment	14	Describe any methods used to assess risk of bias due to missing results in a synthesis (arising from reporting biases).	NA
Certainty assessment	15	Describe any methods used to assess certainty (or confidence) in the body of evidence for an outcome.	NA
**RESULTS**			
Study selection	16a	Describe the results of the search and selection process, from the number of records identified in the search to the number of studies included in the review, ideally using a flow diagram.	6
	16b	Cite studies that might appear to meet the inclusion criteria, but which were excluded, and explain why they were excluded.	Suppl table S3
Study characteristics	17	Cite each included study and present its characteristics.	Suppl table S3
Risk of bias in studies	18	Present assessments of risk of bias for each included study.	NA
Results of individual studies	19	For all outcomes, present, for each study: (a) summary statistics for each group (where appropriate) and (b) an effect estimate and its precision (e.g. confidence/credible interval), ideally using structured tables or plots.	NA
Results of syntheses	20a	For each synthesis, briefly summarise the characteristics and risk of bias among contributing studies.	NA
	20b	Present results of all statistical syntheses conducted. If meta-analysis was done, present for each the summary estimate and its precision (e.g. confidence/credible interval) and measures of statistical heterogeneity. If comparing groups, describe the direction of the effect.	NA
	20c	Present results of all investigations of possible causes of heterogeneity among study results.	NA
	20d	Present results of all sensitivity analyses conducted to assess the robustness of the synthesized results.	NA
Reporting biases	21	Present assessments of risk of bias due to missing results (arising from reporting biases) for each synthesis assessed.	NA
Certainty of evidence	22	Present assessments of certainty (or confidence) in the body of evidence for each outcome assessed.	NA
**DISCUSSION**			
Discussion	23a	Provide a general interpretation of the results in the context of other evidence.	12-14
	23b	Discuss any limitations of the evidence included in the review.	14
	23c	Discuss any limitations of the review processes used.	14
	23d	Discuss implications of the results for practice, policy, and future research.	13-14
**OTHER INFORMATION**			
Registration and protocol	24a	Provide registration information for the review, including register name and registration number, or state that the review was not registered.	4
	24b	Indicate where the review protocol can be accessed, or state that a protocol was not prepared.	4
	24c	Describe and explain any amendments to information provided at registration or in the protocol.	NA
Support	25	Describe sources of financial or non-financial support for the review, and the role of the funders or sponsors in the review.	15
Competing interests	26	Declare any competing interests of review authors.	15

Section and Topic	Item #	Checklist item	Location where item is reported
Availability of data, code and other materials	27	Report which of the following are publicly available and where they can be found: template data collection forms; data extracted from included studies; data used for all analyses; analytic code; any other materials used in the review.	15

Supplementary Table S2. Search strategies for biomedical databases

Database	Search string	Number of publications
PubMED	(("Algeria"[Text Word] OR "Bahrain"[Text Word] OR "Djibouti"[Text Word] OR "Egypt"[Text Word] OR "Iran"[Text Word] OR "Iraq"[Text Word] OR "Israel"[Text Word] OR "Jordan"[Text Word] OR "Kuwait"[Text Word] OR "Lebanon"[Text Word] OR "Libya"[Text Word] OR "Morocco"[Text Word] OR "Oman"[Text Word] OR "Qatar"[Text Word] OR "saudi arabia"[Text Word] OR "KSA"[Text Word] OR "Syria"[Text Word] OR "Tunisia"[Text Word] OR "United Arab Emirates"[Text Word] OR "UAE"[Text Word] OR "Yemen"[Text Word])<br>AND<br>("fetal membranes, premature rupture"[MeSH Terms] OR "premature birth"[MeSH Terms] OR "Chorioamnionitis"[MeSH Terms] OR "preterm premature rupture of membranes"[Title/Abstract] OR "PPROM"[Title/Abstract] OR "PROM"[Title/Abstract] OR "pregnancy latency"[Title/Abstract] OR "preterm delivery"[Title/Abstract] OR "preterm birth"[Title/Abstract] OR "Chorioamnionitis"[Title/Abstract] OR "intra amniotic infection"[Title/Abstract])) AND (2000:2025[pdat])	833
SCOPUS	((TITLE-ABS-KEY (premature rapture of membranes) OR TITLE-ABS-KEY (fetal membranes) OR TITLE-ABS-KEY (chorioamnionitis) OR TITLE-ABS-KEY (preterm premature rapture of membranes) OR TITLE-ABS-KEY (PROM) OR TITLE-ABS-KEY (pprom) OR TITLE-ABS-KEY (pregnancy latency) OR TITLE-ABS-KEY (preterm delivery) OR TITLE-ABS-KEY (preterm birth) OR TITLE-ABS-KEY (intra amniotic infection)))<br>AND<br>((TITLE-ABS-KEY (Algeria) OR TITLE-ABS-KEY (bahrain) OR TITLE-ABS-KEY (djibouti) OR TITLE-ABS-KEY (egypt) OR TITLE-ABS-KEY (Iran) OR TITLE-ABS-KEY (iraq) OR TITLE-ABS-KEY (israel) OR TITLE-ABS-KEY (Jordan) OR TITLE-ABS-KEY (kuwait) OR TITLE-ABS-KEY (lebanon) OR TITLE-ABS-KEY (Libya) OR TITLE-ABS-KEY (morocco) OR TITLE-ABS-KEY (oman) OR TITLE-ABS-KEY (Qatar) OR TITLE-ABS-KEY (saudi arabia) OR TITLE-ABS-KEY (ksa) OR TITLE-ABS-KEY (Syria) OR TITLE-ABS-KEY (tunisia) OR TITLE-ABS-KEY (United Arab Emirates) OR TITLE-ABS-KEY (uae) OR TITLE-ABS-KEY (yemen))) AND PUBYEAR > 1999 AND PUBYEAR < 2026	1932
Embase	'algeria'/exp OR 'algeria' OR 'bahrain':ti,ab,kw OR djibouti:ti,ab,kw OR egypt:ti,ab,kw OR iran:ti,ab,kw OR 'iraq':ti,ab,kw OR israel:ti,ab,kw OR jordan:ti,ab,kw OR kuwait:ti,ab,kw OR lebanon:ti,ab,kw OR libya:ti,ab,kw OR morocco:ti,ab ,kw OR oman:ti,ab,kw OR qatar:ti,ab,kw OR 'saudi arabia':ti,ab,kw OR ksa:ti,ab,kw OR syria:ti,ab,kw OR tunisia:ti,ab,kw OR 'united arabemirates':ti,ab,kw OR uae:ti,ab,kw OR yemen:ti,ab,kw<br>AND	589

	'premature rupture of membranes'/exp OR 'premature rupture of membranes' OR 'premature birth':ti,ab,kw OR 'chorioamnionitis':ti,ab,kw OR 'premature labor':ti,ab,kw OR 'preterm rupture of membranes':ti,ab,kw OR pprom:ti,ab,kw OR prom:ti,ab,kw OR 'pregnancy latency':ti,ab,kw OR 'intra amniotic infection':ti,ab,kw<br>Filter: articles published from 2000	
Web c scienc	Algeria (Topic) or Bahrain (Topic) or Djibouti (Topic) or Egypt (Topic) or Iran (Topic) or Iraq (Topic) or Israel (Topic) or Jordan (Topic) or Kuwait (Topic) or Lebanon (Topic) or Libya (Topic) or Morocco(Topic) or Oman (Topic) or Qatar (Topic) or Saudi Arabia(Topic) or KSA (Topic) or Syria (Topic) or Tunisia (Topic) or United Arab Emirates (Topic)or UAE (Topic) or Yemen (Topic) and Preprint Citation Index (Exclude – Database)<br>AND<br>(((((((TS=(premature rapture of membrane)) OR TS=(preterm premature rapture of membrane)) OR TS=(premature birth)) OR TS=(premature delivery)) OR TS=(premature labour)) OR TS=(PROM)) OR TS=(PPROM)) OR TS=(pregnancy latency)and Preprint Citation Index (Exclude – Database)<br>Filter: articles published from 2000	2006

Supplementary Table S3. Characteristics of included studies

Author, year	Country	Sample size	Study design	Statistical approach	Rupture type	Study focus
Abdelazim 2012 (1)	Kuwait	150	Cross-sectional	Chi-square test; Un-paired Student (t) test; Positive Predictive Value and Negative predictive Value.	PROM	Confirmation of PROM with PAMG-1, Ferning, and Nitrazine test
Abdelraheim 2019 (2)	Egypt	1708	Case-control	Chi-square or Fisher exact test; Independent sample T test; univariate and multivariate logistic regression analyses.	PROM	Intraabdominal infection following CS. Case and control groups were compared based on different clinical characteristics including PROM
Abdollahi 2014 (3)	Iran	1459	Cohort	Prevalence distribution; simple logistic regression; multiple logistic regression	PROM	Impact of psychological violence on pregnancy outcomes including PROM

Abdollahi 2015 (4)	Iran	1461	Cohort	Prevalence; Chi-Square test or Fisher's Exact test; logistic regression model; multivariate model.	PROM	Impact of physical violence on pregnancy outcomes including PROM
Abdulhussain 2022 (5)	Iraq	100	RTC	Independent sample t-test: Mann Whitney U U-test; Chi-square test	PPROM	Vitamin C supplementation for prevention of PPROM in women with history of PPROM
Abedzadeh-Kalahroudi 2015 (6)	Iran	7154	Cross-sectional	Independent-sample t-test; chi-square test; Multiple logistic regression.	PROM	Neonal birth injuries due to pregnancy complications including PROM
Afjeh 2013 (7)	Iran	4629	Cross-sectional	Chi-square; ANOVA	PROM	Risk factors (including PROM) for Neonatal Resuscitation
Afkhamzadeh 2021(8)	Iran	1025	Cohort	Chi-square; Fisher's exact test; independent sample t-test; incidence rate; relative risk.	PROM	Impact of intimate partner violence on pregnancy outcomes including PROM
Al-Jishi 2013 (9)	Saudi Arabia	638	Cohort	N/A	PPROM	Perinatal outcomes (including PPROM) of placing cervical cerclage
Al-Kadri 2013 (10)	Saudi Arabia	299	Case-control	Bivariate analyses; Chi-square; Student's t-test; Stepwise multiple logistic regression.	PROM	Maternal risk factors (including PROM) for early-onset group B streptococcal disease
Al-Riyami 2013 (11)	Oman	44	Cohort	NA	PPROM	Perinatal outcomes in women with mid-term PROM
Al-Shehab 2025 (12)	Yemen	97	Cross-sectional	Pearson's chi-square; Fisher's exact test; independent samples t-test;	PROM	Risk factors for early- and late-onset sepsis in neonates

Al-Talib 2013 (13)	Iraq	398	Cross-sectional	Chi-square; Fisher's exact test	PROM	PROM as one of risk factors for neonatal Septicemia
Al-Wassia 2017 (14)	Saudi Arabia	284	Case-control	t-test; Mann-Whitney U tests; Fisher's exact tests; multivariate logistic regression;	PROM	Risk factors for admission of infants to NICU
Alavi 2021 (15)	Iran	400	Case-control	conditional logistic regression; descriptive statistics	PROM	Predictors of preterm birth
Albahlol 2020 (16)	Saudi Arabia	322	Cross-sectional	One-way ANOVA with Tukey's post-test; Spearman correlation coefficient test; ROC AUC.	PROM	Vitamin D Status and Pregnancy Complications
Alfarwati 2019 (17)	Saudi Arabia	118	Cross-sectional	t-test; Chi-square	PROM	Risk factors for respiratory distress syndrome
AlGhadeer 2024 (18)	Saudi Arabia	400	Prevalence study	Frequency; Pearson chi-square test; exact probability test	PROM	Infant Complications of diabetic mothers and maternal obstetric profile
Alhainiah 2018 (19)	Saudi Arabia	415	Cross-sectional	N/A	PROM	Pregnancy outcomes in grand multipara women
Alhasoon 2024 (20)	Saudi Arabia	256	Case-control	Descriptive statistics; Chi-square test; univariate and multivariate regression models; survival analyses	PROM	Survival rates of extremely low-birth-weight Infants. PROM longer tan 18 hrs was used for characteristics of study cohort
Ali 2012 (21)	Saudi Arabia	102	Cross-sectional	Student’s t-test; Chi-square test; Fisher’s test; analysis of variance	PROM	Transmission of *Candida* colonization from mothers to infants. Duration of PROM was correlated with *Candida* colonization

Ali 2017 (22)	Saudi Arabia	38	Case-control	ROC; Chi-square test	PROM	Role of serum apelin as a diagnostic tool in retinopathy of prematurity. PROM was a risk factor of the disease
Ali 2024 (23)	Iraq	90	Case-control	Shapiro-Wilk test; ANOVA; Kruskal-Wallis test; Fisher-Freeman-Halton exact tests; ROC AUC.	PPROM	Diagnostic Accuracy ofEotaxin-2 as a Marker for PPROM
Alijahan 2014 (24)	Iran	935	Case-control	Chi-square; univariate and multivariate logistic regression	PROM	PROM as a risk factor for preterm birth
Al Jahdali 2022 (25)	Saudi Arabia	4318	Cohort	Chi-square; Fisher exact test	PROM	Maternal age as a predictor of PROM.
Al Jama 2012 (26)	Saudi Arabia	447	Case-control	Chi-square; Fisher exact test; Student 't' test; multivariate logistic regression.	PROM	PROM longer than 8 hours as a risk factor for wound infection following lower segment CS
Almaghrabi 2022 (27)	Saudi Arabia	1201	Case-control	Descriptive statistical analyses; T-test; Chi-square	PROM	GBS Colonization as a risk factor for PROM
Almaghrabi_1 2022 (28)	Saudi Arabia	1201	Cross-sectional	T-test; Chi-square	PROM and PPROM	Microbial profile of women with PPROM and PROM; neonatal death following rupture of membranes.
AlMatary 2017 (29)	Saudi Arabia	86	Cross-sectional	Chi-square and odds ratio; binary logistic regression for survival analysis; multiple logistic regression model	PROM	PROM as a risk factor for neonatal Pneumothorax and its outcomes.
Almuneef 2000 (30)	Saudi Arabia	23	Cross-sectional	Chi-square test; Fisher’s Exact Test	PROM	PROM as a risk factor for GBS

AlRiyami 2013 (31)	Oman	44	Cohort	Multivariable logistic regression	PPROM	Risk factors, maternal and neonatal outcomes of extreme preterm PROM
Amini 2008 (32)	Iran	198	Cross-sectional	Chi-square	PROM	PROM was used as a part of descriptive statistics in children with ophthalmia neonatorum due to Staphylococcal or E.coli infection
Aminisani 2009 (33)	Iran	179	RTC	Chi-square; Fishers' exact test; ANOVA	PROM	Zink supplementation for prevening law birth weight in infants. PROM is used as a part of descriptive statistics.
Aryavand 2024 (34)	Iran	619	Cohort	T-test; ANOVA; the Chi-square	PROM	Concentration of Fetal fraction in cell-free DNA as a predictor of adverse pregnancy outcomes including PROM.
Asgarian 2020 (35)	Iran	602	Cross-sectional	Chi-square test	PROM	Law birth weight in infants. PROM used as part of cohort characteristics.
Asindi 2002 (36)	Saudi Arabia	97	Cohort	N/A	PROM and PPROM	Bacterial pathogens involved in maternal and neonatal colonization, and the major bacterial pathogens of neonatal sepsis in PROM.
Azargoon 2006 (37)	Iran	263	Cohort	Chi-square; Student's t-test	PPROM	Bacterial vaginosis, Trichomonas vaginalis, and vaginal acidity as risk factors for PPROM.
Azizieh 2015 (38)	Kuwait	95	Case-control	Mann-Whitney test	PROM	Levels of tumor necrosis factor-α as a risk factor for pregnancy complications including PROM.
Bener 2012 (39)	Qatar	1674	Cross-sectional	Student's t-test; Fisher Exact test; Chi-Square test; univariate logistic regression; multiple logistic regression	PROM	PROM as a risk factor for low birth weight
Bhakta 2021 (40)	Saudi Arabia	217	Cross-sectional	Chi-square test; unpaired t-test	PROM	Pregnancy Outcomes of bacterial vaginosis (including PROM).

Borna 2010 (41)	Iran	328	Case-control	Chi-square test; Fisher exact test; Student t test; Stepwise multiple logistic regression;	PROM	PROM as one of predictors of fetal injury
Boskabadi 2011(42)	Iran	150	Cross-sectional	Student T-test; Chi-square test	PROM	Neonatal Complications due to Prolonged ROM
Boskabadi 2022 (43)	Iran	213	Cross-sectional	Chi-Square; t-test	PROM	PROM as part of cohort characteristics of preterm babies with and without Retinopathy of Prematurity born to mothers with and without preeclampsia
Bouzari 2018 (44)	Iran	120	Cross-sectional	Chi-square; T-test	PROM	Vaginal discharge concentrations of β-human chorionic gonadotropin, creatinine, and urea for diagnosing PROM
Broumand 2018 (45)	Iran	48	Cohort	descriptive statistics; independent t-test; Pearson correlation coefficient	PROM and PPROM	Predictive Values of Maternal Serum Levels of Procalcitonin, ESR, CRP, and WBC in the Diagnosis of Chorioamnionitis in Mothers with Preterm Premature Rupture of Membrane
DahmanHAB 2020 (46)	Yemen	500	Case-control	Mann-Whitney U-test; Chi-square test; OR; Fisher's exact test; binary logistic regression	PROM	Risk factors for preterm birth including PROM
Darine 2021 (47)	Tunisia	251	Case-control	Odds ratio	PROM	Genetal tract infection as a risk factor for PROM
Derakhshi 2014 (48)	Iran	600	Case-control	T-test, Mann–Whitney U test; Chi-square test	PROM	PROM among risk factors for preterm labour
Ebadi 2025 (49)	Iran	913	RTC	independent t-tests; chi-square tests; repeated-measures ANOVA	PROM	Comparison of protocols for labour induction in woment with PROM

Ebrahimi 2021 (50)	Iran	345	Cross-sectional	Fisher's exact test; Chi-square test; Kruskal-Wallis test; ANOVA, and Bonfrroni post hoc test; Spearman correlation test; multiple linear regression analysis	PROM	Adverse pregnancy outcomes (including PROM) in women of different age
Elbaradie 2009 (51)	Egypt	95	Cross-sectional	Mann Whitney U; Chi square test	PPROM	PPROM as part of cohort characteristics of women with and without GBS
ElSawi 2013 (52)	Egypt	100	Cross-sectional	Student t-test; Chi-square test; Pearson's correlation	PPROM	lead concentration in Umbilical Cord-blood as risk factor for pregnancy outcomes including PPROM
ElTaher 2004 (53)	Qatar	150	Case-control	relative risk; Chi-square test; odds ratio	PROM and PPROM	Level of CRP in women with PROM and PPROM
Eltayeb 2010 (54)	Egypt	35	Cross-sectional	hypothesis test for two proportions from independent groups	PROM	PROM as cohort characteristics in cases with and without necrotizing entercolitis
Fallatah 2019 (55)	Saudi Arabia	1337	Cross-sectional	Independent t-test; correlation test; chi-square test; binary and multinomial logistic regression	PROM and PPROM	Obesity class as a risk factor for PROM/PPROM and other pregnancy complications
Faramarzi 2005 (56)	Iran	3275	Cross-sectional	prevalence odds ratios; logistic regression	PROM	Phsycial, sexual and emotional domestic violence as a risk factor for PROM and other pregnancy complications
Farshad 2021 (57)	Iran	299	Cohort	a path analysis	PROM and PPROM	Periodontal disease and tooth brushing habits as a risk factor for PROM/PPROM
Ghardallou 2019 (58)	Tunisia	500	Cross-sectional	descriptive data analysis	PROM	Management of pregnancy; PROM as a reason for referral to a territory maternity care

Ghomian 2013 (59)	Iran	170	RTC	T-test; Mann-Whitney; Chi-square test	PROM and PPROM	Prevention of PROM/PPROM with vitamin C supplementation
Gomaa 2021 (60)	Egypt	2328	Case-control	T-test; Chi-square test; Fisher exact test	PROM	Risk factors for surgical site infection after including PROM
Gouda 2025 (61)	Egypt	100	Cross-sectional	Chi-square test; Pearson's correlation coefficient	PPROM	Risk factors for PPROM
Hadavi 2011 (62)	Iran	642	Case-control	Chi-suqare test; independent sample t test	PROM	PROM as one of risk factors for perinatal mortality
HaghshenasMojaveri 2021 (63)	Iran	828	Case-control	Univariate logistic regression; multivariate logistic regression; Bayesian logistic regression	PROM	PROM as one of risk factors for retinopathy of prematurity
HalimiAsl 2017 (64)	Iran	810	Cross-sectional	Chi-square test; Fisher's exact test	PROM	Risk factors for PROM
Hamta 2017 (65)	Iran	431	Case-control	Propensity Score Matching Analysis; Student's t- test; Restricted iterative generalized least square; F-statistic; Inter-Class Correlation	PROM	PROM as part cohort characteristics of women with cervical cerclare
Hijazi 2023 (66)	Jordan	630	Cohort	Chi-square test; ANOVA; Binary logistic regression model	PROM	Prolonged rapture of membrane as a risk factor for preterm birth and low birth weight
Hussein 2021 (67)	Saudi Arabia	650	Cross-sectional	Independent samples t-test; sensitivity, specificity, positive predictive values,	PPROM	Maternal CRP and platelet volume as predictors of PPROM

				negative predictive values, likelihood ratios, and odds ratios		
Ibrahim 2015 (68)	Egypt	1857	Case-control	Student t-test; Chi-square; Relative risk	PROM	Intimate partner violence as a risk factor for pregnancy complications including PROM
Jafarpour 2025 (69)	Iran	660	Case-control	Conditional logistic regression test	PROM	PROM as a one of risk factors for prematurity
Jahromi 2011 (70)	Iran	327	Cross-sectional	Kruskal-Wallis test; Mann-Whitney U test; Fisher's exact test	PROM	Preterm spontaneous uterine contractions as a risk factor for pregnancy complications including PROM
Jalil 2019 (71)	Bahrain	2117	Cohort	two-sided unpaired T-test, Mann-Whitney U test, the Chi-square test, the Fisher-Freeman-Halton exact test	PROM	GBS as a risk factor for PROM and other pregnancy complications
Kalantari 2010 (72)	Iran	24	Cross-sectional	Pearson correlation coefficient	PPROM	The relationship between myometrial thickness and the latency in PPROM
Kariman 2011 (73)	Iran	181	Diagnostic test accuracy	ROC; Chi Square test; student's test; Mann-withney test	PROM	Comparison of methods of diagnostics of PROM
Kariman 2012 (74)	Iran	114	Diagnostic test accuracy	ROC; Chi-square test; Mann Withney test; T-test	PROM	Vaginal prolacting for diagnostics of PROM
Kashanian 2018 (75)	Iran	231	RTC	Chi-square test; Fisher's exact test; independent samples T test; Mann–Whitney test; binary logistic regression model; relative risk	PROM and PPROM	Copper supplementation for prevention of PROM
Khademolkhamseh 2022 (76)	Iran	90	RTC	Chi-square test; exact Chi-square	PROM	The impact of equisetum arvense vaginal cream on PROM in

				test; independent t-test; Mann-Whitney test		primiparous women with striae gravidarum
Khalessi 2014 (77)	Iran	20	Cross-sectional	Descriptive statistics	PROM	PROM as one of risk factors for neonatal meningitis
Khanghah 2020 (78)	Iran	394	Cross-sectional	Chi-square test; Fisher's exact test; Student's t-test; Mann-Whitney test	PROM	Depression as a risk factor for pregnancy complications including PROM
Khasawneh 2020 (79)	Jordan	2595	Cohort	Chi-square test; backward conditional logistic regression; adjusted odds ratios	PROM	PROM as one of risk factors of NICU admission after CS
Khasawneh 2020 (80)	Jordan	1444	Cross-sectional	Pearson Chi-square test; Student's t-test; ANOVA; posthoc residual analysis; Fisher's least significant difference test	PROM	PROM as one of risk factors for NICU admission
Khezri 2025 (81)	Iran	387	Case-control	Univariable and multivariable logistic regression	PROM	PROM as one of risk factors for preterm birth
Khezri 2025 (82)	Iran	414	Case-control	Univariable and multivariable logistic regression analyses	PROM	PROM as one of risk factors for preterm birth
Khodakarami 2009 (83)	Iran	313	Cross-sectional	Chi-squared analysis; logistic regression; multivariate logistic regression analysis	PROM	Abuse as a risk factor for pregnancy complications
Khoigani 2012 (84)	Iran	520	Cohort	Student's t-test; Pearson correlation	PPROM	Hemoglobin and hematocrit as risk factor for pregnancy complications

				test; regression analysis		
Kouhkan 2021 (85)	Iran	531	Cohort	T-test; Mann-Whitney test; Chi-square test; ordinal logistic regression model	PROM	Gestational diabetes mellitus as a risk factor for pregnancy complications
Maher 2013 (86)	Saudi Arabia	502	RTC	Chi-squared test; Fisher's exact test; Student's t-test	PROM	Prevention of preterm birth with progesterone. PROM is used as a cohort characteristic
Mirghani 2010 (87)	Saudi Arabia	176	Cross-sectional	Student's t-test; Chi-square test	PROM	Lead exposure as a risk factor for pregnancy complications
Mirzamoradi 2014 (88)	Iran	92	RTC	Student t test; Mann-Whitney test; regression analysis	PPROM	Magnesium sulfate supplementation for delaying the active phase of labour in women with PPROM
Moghaddam 2025 (89)	Iran	120	Cross-sectional	T-test; Chi-square test; logistic regression	PROM	PROM longer than 6 hrs as a obstetric characteristic of women with CS
MoghaddamBanaem 2012 (90)	Iran	797	Cohort	Mann–Whitney U-test; binary logistic regression model; ROC analysis; sensitivity, specificity; likelihood ratios	PPROM	Maternal CRP in early pregnancy as predictor of PPROM
Mohammed 2022 (91)	Iraq	700	Case-control	Chi-square test; odds ratios; relative risk; binary logistic regressions	PROM	PROM as one of risk factors for prematurity
Nabhan 2014 (92)	Egypt	1640	RTC	Risk ratios; mean difference; T-test; Fisher exact test	PROM	Antibiotic prophylaxis in PROM
Nair 2024 (93)	Oman	142	Case-control	Chi-square test; Odds ratio	PPROM	Maternal and neonatal outcomes of PPROM in women with and without cervical cerclage

Najati 2009 (94)	Iran	100	Case-control	Descriptive; student t-test	PROM	Umbilical cord interlukin-8 in low birth weight born to mothers with and without PROM
Najjarzadeha 2022 (95)	Iran	371	Cohort	Multivariable binary logistic regression with the backward stepwise strategy	PPROM	PPROM as a risk factor for prematurity
NamavarJahromi 2008 (96)	Iran	1197	Case-control	Student t-test; Chi-square test; Fisher's exact test	PROM and PPROM	GBS as a risk factor for PROM/PPROM
Nasab 2021 (97)	Iran	932	Case-control	Chi-square test; Fisher's exact test; independent samples t-test; multivariate logistic regression model	PROM	PROM as one of risk factors for perinatal mortality
Naseh 2020 (98)	Iran	54	Cross-sectional	T-test; Chi-square test	PROM	PROM as a risk factor for a serious bacterial infections among newborns.
Nazarpour 2022 (99)	Iran	1843	Cohort	Pearson's Chi-square test; independent samples t-test; Mann–Whitney U test; generalized linear regression model; odds ratio; mean difference	PROM and PPROM	Isolated maternal hypothyroxinemia as a risk factor for pregnancy complications
Neamah 2022 (100)	Iraq	90	Cohort	Descriptive analysis; logistic regression analysis; Odds ratio	PROM	PROM as a risk factor for retinopathy of prematurity
Niyaty 2021 (101)	Iran	203	Cohort	Independent Student's t-test; Chi-square test; Fisher's exact test; logistic regression analysis.	PPROM	Maternal metabolic syndrome and lipid profile as a risk factor for PPROM

Nojomi 2010 (102)	Iran	538	Cross-sectional	T-test; Multiple logistic regression analysis	PROM	Age are a risk factor for pregnancy complications
Ouladsahebmadarek 2011 (103)	Iran	782	RTC	T-test; Mann–Whitney U test; isher exact test; Chi-aquare test	PROM	Withholding iron supplementation and its affect on pregnancy outcomes
Panahi 2025 (104)	Iran	396	Cross-sectional	Fisher's exact test; Chi-square test; Mann-Whitney U-test; Kruskal-Wallis test	PPROM	Perinatal outcomes of PPROM
ParaparambilVellamgot 2025 (105)	Qatar	157	Cohort	Logistic regression model; Chi-square test; Fisher's exact test; Mann-Whitney U-test.	PROM	PROM longer than 18 hours as one of risk factors for GBS related early onset sepsis
Rad 2022 (106)	Iran	336	Cross-sectional	Independent t-test; Chi-square test; crude and adjusted logistic regression models	PPROM	Maternal and neonatal outcomes of PPROM and latency
Rahmanian 2014 (107)	Iran	150	Cross-sectional	Chi-square test; Kruskal-Walis test; One-way analysis of variance and Tukey tests	PROM	Serum copper and zinc levels as risk factor for PROM
Rejali 2017 (108)	Iran	940	Case-control	Chi-square test; Fisher’s exact test; likelihood ratio; Student’s t-test; logistic regression model	PROM	PROM as an obstetric characteristic of deliveries with low and normal birth weight infants
Sadat Mousavi 2018 (109)	Iran	91	Cross-sectional	Independent t-test; Chi square	PPROM	AFI as a predictor of meternal and neonatal outcomes of PPROM

Salama 2023 (110)	Egypt	522	Case-control	Chi-square test; multivariate regression	PROM	PROM as one of risk factors for neonatal sepsis
Saleh-Gargari 2009 (111)	Iran	900	Case-control	T-test; Chi-square test; Fisher's exact test; odds ratios; Multinomial logistic regression	PROM and PPROM	Maternal HBsAg status as a risk factor for pregnancy complications
Sehhati-Shafaii 2013 (112)	Iran	960	Cross-sectional	Chi-square; T-test; logistic regression model	PROM	PROM as one of risk factors for preterm labor
Seoud 2010 (113)	Lebanon	775	Cross-sectional	Bivariate analysis; chi-squared test; Student's t-test	PROM	PROM as one of risk factors for GBS
Shahgeibi 2009 (114)	Iran	533	Case-control	Chi-square test; Fisher exact; Yates correction	PROM	Bacterial vaginosis as risk factor for pregnancy complications
Shahnazi 2017 (115)	Iran	92	RTC	Chi-square test; trend test; exact test; t-test; Mann-Whitney test	PROM and PPROM	Zinc supplementation for prevention of PROM and PPROM
Sharami 2021 (116)	Iran	300	Case-control	Chi-square test; the Mann-Whitney U test; independent sample t-test; receiver-operator curve	PROM	Association between platelets/lymphocyte ratio and PROM
Sobhani 2018 (117)	Iran	402	Cross-sectional	Chi-square test; multiple logistic regression	PROM	Domestic violence as a risk factor for pregnancy complications
Surrati 2024 (118)	Saudi Arabia	64	Cross-sectional	Chi-square test	PROM	PROM as one of risk factors for neonatal GBS infection
Tabatabaei 2011 (119)	Iran	5172	Cross-sectional	Chi-square; Multiple logistic regression; odds ratios	PPROM	Gestation weight gain and pregnancy BMI as risk factors for pregnancy complications
Taghavi 2009 (120)	Iran	99	Case-control	Student's t-test; Mann–Whitney U-	PPROM	Neonatal CRP as a predictor of outcomes after PPROM

				test; chi-test or Fisher’s exact test; Mantel–Haenszel chi-test; univariate analyses; odds ratios		
Tahmasebifard 2025 (121)	Iran	3639	Case-control	univariate and multiple (multivariable) logistic regression models; adjusted odds ratio	PPROM	Vaginal bleeding as a risk factor for PPROM and other pregnancy complications
Tajeran 2024 (122)	Iran	151	Cohort	t-test; Chi-square test; Receiver Operating Characteristic curve; Sensitivity; specificity; positive predictive value; negative predictive value	PROM	Cervical parameters as predictors of pregnancy outcomes
Tara 2010 (123)	Iran	125	RTC	Fisher’s exact test; paired samples t-test; Wilcoxon test	PROM	Selenium supplementation for prevention of PROM
Tavassoli 2010 (124)	Iran	137	Cohort	Chi-square test; T-test; Mann-Whitney test; Kruskal Wallis test	PPROM	Impact of amniotic fluid index on pregnancy outcomes
Thabet 2025 (125)	Yemen	505	Cross-sectional	Fisher’s exact test; binary logistic regression; multivariable logistic regression; adjusted odds ratios; odds ratios	PROM	PROM as one of indication for labour induction and its outcome
Vaezi 2017 (126)	Iran	580	Case-control	T-test; chi-square test; Man-Whitney U-test; logistic	PROM	Maternal asthma as a risk factor for pregnancy complications

				regression model; crude and adjusted odds ratio		
Vanda 2024 (127)	Iran	114	RTC	Chi-square; independent t-test	PROM and PPROM	Impact of oral probiotic in women with cerclage on pregnancy outcomes
Vellamgot 2022 (128)	Qatar	415	Cross-sectional	Chi-square test; Fisher's Exact test; unpaired t-test; ANOVA	PROM	Short term neonatal outcomes after clinical chorioamnionitis
Wahabi 2024 (129)	Saudi Arabia	1894	Case-control	chi-square test; Fisher's Exact test; ANOVA; Logistic regression	PROM and PPROM	Predictors and outcomes of PROM and PPROM
Yazdani 2015 (130)	Iran	231	Case-control	Chi-square test; Fisher-exact test; t-test; logistic regression	PROM	Correlation of pregnancy outcome with quadruple screening test
Younesi 2023 (131)	Iran	9773	Cohort	Chi-2 test; ANOVA test	PROM	Extreme βHCG levels in first trimester as a risk factor for pregnancy complications
ZahiriSorouri 2016 (132)	Iran	540	RTC	two-tailed independent t-test; Chi-square; Fisher's exact test	PROM	The effect of zinc supplementation on pregnancy outcomes
Zamani 2013 (133)	Iran	60	RTC	Student t-test; chi-square test; Fisher exact test	PPROM	Effects of ascorbic acid on serum level of unconjugated estriol and its relationship with PPROM
Zamzami 2006 (134)	Saudi Arabia	344	Case-control	Student's t-test; Chi-square test	PROM	Labour management in PROM
Ziadeh 2002 (135)	Jordan	291	Cross-sectional	Student's t-test; Chi-square test; Mann-Whitney U-test	PROM	Advanced maternal age as a risk factor for pregnancy complications
Ziaei 2006 (136)	Iran	299	Case-control	Chi-square test; odds ratio	PROM	Bacterial vaginosis as a risk factor for PROM

Supplementary Table S4. Critical appraisal of cross-sectional studies

Author, year	1.Was the research question or objective in this paper clearly stated?	2.Was the study population clearly specified and defined?	3.Was the participation rate of eligible persons at least 50%?	4.Were all the subjects selected or recruited from the same or similar populations (including the same time period)? Were inclusion and exclusion criteria for being in the study prespecified and applied uniformly to all participants?	5.Was a sample size justification, power description, or variance and effect estimates provided?	6.For the analyses in this paper, were the exposure(s) of interest measured prior to the outcome(s) being measured?	7.Was the timeframe sufficient so that one could reasonably expect to see an association between exposure and outcome if it existed?	8. For exposures that can vary in amount or level, did the study examine different levels of the exposure as related to the outcome (e.g., categories of exposure, or exposure measured as continuous variable)?	9. Were the exposure measures (independent variables) clearly defined, valid, reliable, and implemented consistently across all study participants?	10. Was the exposure(s) assessed more than once over time?	11. Were the outcome measures (dependent variables) clearly defined, valid, reliable, and implemented consistently across all study participants?	12. Were the outcome assessors blinded to the exposure status of participants?	13. Was loss to follow-up after baseline 20% or yes?	14. Were key potential confounding variables measured and adjusted statistically for their impact on the relationship between exposure(s) and outcome(s)?
Mousavi, 2018	Yes	Yes	Yes	Yes	No	NA	Yes	NA	Yes	No	Yes	No	Yes	No
Abdelazim, 2011	Yes	Yes	Yes	Yes	No	NA	Yes	NA	Yes	No	Yes	No	Yes	No
Abedzadeh-Kalahroudi, 2015	Yes	Yes	Yes	Yes	No	Yes	Yes	Yes	Yes	No	Yes	No	Yes	Yes
Afjeh SA, 2013	Yes	Yes	Yes	Yes	No	Yes	Yes	Yes	Yes	No	Yes	No	Yes	Yes
Abdollahi, 2014	Yes	Yes	Yes	Yes	No	Yes	Yes	Yes	Yes (but some recall and report bias may be possible)	No	Yes	No	Yes	Yes
Albahlol, 2020	Yes	Yes	Yes	Yes	No	NA	Yes	NA	Yes	No	Yes	No	Yes	No
Alfarwati, 2019	Yes	Yes	Yes	Yes	No	NA	Yes	NA	Yes	No	Yes	No	Yes	No
Abdollahi, 2015	Yes	Yes	Yes	Yes	No	Yes	Yes	Yes	Yes	No	Yes	No	Yes	Yes
Alhainiah, 2018	Yes	Yes	Yes	Yes	No	NA	Yes	NA	Yes	No	Yes	No	Yes	No
Afkhamzadeh, 2021	Yes	Yes	Yes	Yes	No	No	Yes	No	Yes	No	Yes	No	Yes	Yes
AlGhadeer, 2024	Yes	Yes	Yes	Yes	No	NA	Yes	Yes	Yes	Yes	Yes	No	Yes	No
Almaghrabi 2022	Yes	Yes	Yes	Yes	No	No	Yes	Yes	Yes	No	Yes	No	Yes	No
Al-Jishi 2013	Yes	Yes	Yes	Yes	No	Yes	Yes	No	Yes	No	Yes	No	Yes	No
Ali 2012	Yes	Yes	Yes	Yes	No	Yes	Yes	No	Yes	Yes	Yes	No	Yes	Partially
AlJahdali 2022	Yes	Yes	Yes	Yes	No	Yes	Yes	Yes	Yes	No	Yes	No	yes	Partially
Al-Riyami 2013	Yes	Yes	Yes	Yes	No	Yes	Yes	NA	Yes	No	Yes	No	Yes	Yes
Bhakta 2021	Yes	Yes	Yes	Yes	No	Yes	Yes	No	Yes	No	Yes	No	Yes	Yes
AlMatary 2017	Yes	Yes	Yes	Yes	No	NA	Yes	No	Yes	No	Yes	No	yes	Partially

Almuneef 2000	Yes	Yes	Yes	Yes	No	Yes	Yes	No	Yes	Yes	Yes	No	Yes	No
AlRiyami 2013	Yes	Yes	NR	Yes	No	Yes	Yes	Partially	Yes	No	Yes	No	NA	Partially
Al-Shehab 2025	Yes	Yes	Yes	Yes	No	Yes	Yes	Partially	Yes	No	Yes	No	NA	No
Younesi 2023	Yes	Yes	Yes	Yes	No	Yes	Yes	Yes	Yes	No	Yes	No	Yes	Partially
Nojomi 2010	Yes	Yes	Yes	Yes	No	Yes	Yes	Yes	Yes	No	Yes	No	Yes	Yes
Niyaty 2021	Yes	Yes	Yes	Yes	Yes	Yes	Yes	Yes	Yes	No	Yes	No	Yes	Yes
Khoigani 2012	Yes	Yes	Yes	Yes	No	Yes	Yes	Yes	Yes	Yes	Yes	No	Yes	Yes
Hijazi 2023	Yes	Yes	Yes	Yes	No	Yes	Yes	Yes	Yes	No	Yes	No	Yes	Yes
Zamzami 2006	Yes	Yes	Yes	Yes	No	Yes	Yes	Partially	Yes	No	Yes	No	Yes	Partially
Kariman 2012	Yes	Yes	NR	Yes	no	Partially	Yes	Yes	Yes	No	Yes	No	Yes	NA
Tajeran 2024	Yes	Yes	NR	Yes	No	Yes	Yes	Yes	Yes	No	Yes	No	Yes	No
Bouzari 2018	Yes	Yes	NR	Yes	Yes	No	Yes	Yes	Yes	No	Yes, mostly	No	Yes	NA
Asindi 2002	Yes	Yes	Yes	Yes	No	Yes	Yes	No	Yes	No	Yes	No	Yes	No
Boskabadi 2011	Yes	Yes	Yes	Yes	No	Yes	Yes	Partially yes	Yes	No	Yes, mostly	No	Yes	No
Kariman 2011	Yes	Yes	NR	Yes	Yes	Partially	Yes	Yes	Yes	No	Yes	No	Yes	NA
Asgarian 2020	Yes	Yes	Yes	Yes	No	Yes	Yes	Partially yes	Yes	No	Yes	No	Yes	No
Amini 2008	Yes	Yes	Yes	No	Yes	Yes	Partially yes	Yes	No	Yes	No	Yes	No	Yes
Naseh 2020	Yes	Yes	NR	Yes	No	Yes	Yes	No	CD	No	Yes	No	Yes	No
Bener 2012	Yes	Yes	Yes	Yes	No	Yes	Yes	Partially yes	Yes	No	Yes	No	Yes	Yes
Broumand 2018	Yes	Yes	NR	Partially	No	Yes	Yes	Yes	Yes	Yes	Yes	NR	Yes	No
Eltayeb 2010	Yes	Yes	NR	Yes	No	Yes	Yes	Yes	Partially	No	Yes	No	Yes	No
Hussein 2021	Yes	Yes	NR	Yes	No	Yes	Yes	Yes	Yes	No	Yes	NR	NR	No
Kalantari 2010	Yes	Yes	CD	Yes	partially (no power analysis or expected effect size justification)	Yes	Yes	Yes	Yes	No	Yes	No	Yes	No
ElSawi 2013	Yes	Yes	NR	Yes	No	Yes	Yes	Yes	Yes	No	Yes	No	Yes	No
Ziadeh 2002	Yes	Yes	NR	Yes	No	Yes	Yes	Yes	Yes	No	Yes	No	Yes	NR
Azargoon 2006	Yes	NR	Yes	No	Yes	Yes	Yes	Yes	Yes	Yes	No	NR	No	Yes
Sobhani 2018	Yes	Yes	Yes	Yes	Yes	Partially	Yes	Yes	Yes	No	Yes	No	Yes	Yes
Sehhati-Shafaii 2013	Yes	Yes	NR	Partially	No	Partially	Yes	No	Partially	No	Yes	NR	NA	Partially
Jalil 2019	Yes	Yes	NA	NR	No	Partially	Yes	Yes	Partially	Yes	Yes	No	NR	No
Tabatabaei 2011	Yes	Yes	Yes	Yes	No	Yes	Yes	Yes	Partially	No	Yes	No	Yes	Yes
Ghardallou 2019	Yes	Yes	Yes	No	NA	NA	Yes	Yes	No	Yes	No	Yes	No	Yes
Boskabadi 2022	Yes	Yes	NR	Partially	No	Yes	Yes	No	Yes	No	Yes	No	Yes	No
HalimiAsl 2017	Yes	Yes	NR	Yes	No	Partially	Yes	Yes	Partially	No	Yes	No	Yes	Yes
Khodakarami 2009	Yes	Yes	Yes	No	Yes	Yes	Partially	Yes	No	Yes	No	Yes	Yes	Yes
Thabet 2025	Yes	Yes	NR	Yes	Yes	Yes	Yes	Yes	Yes	No	Yes	No	Yes	Yes
Khasawneh 2020	Yes	Yes	NA	Yes	No	Yes	Yes	Yes	Yes	No	Yes	NR	Yes	No
Neamah 2022	Yes	Yes	No	Yes	No	Yes	Yes	Yes	Partially	No	Yes	NR	Yes	No
Al-Talib 2013	Yes	Yes	NA	Yes	No	Yes	Yes	Yes	Yes	No	Yes	NR	Yes	No
Farshad 2021	Yes	Yes	NR	Yes	Yes	Yes	Yes	Yes	Yes	No	Yes	No	NR	Yes
Elbaradie 2009	Yes	Yes	NR	Yes	No	Yes	Yes	No	Yes	No	Yes	CD	Yes	No
Fallatah 2019	Yes	Yes	No	Yes	No	No	Yes	Yes	Yes	No	Yes	No	Not	Yes

Faramarzi 2005	Yes	Yes	NR	Yes	No	No	Yes	Partially yes	Yes	No	Yes	No	NR	Yes
Rahmanian 2014	Yes	Yes	CD	Yes	yes	Yes	Yes	Yes	Yes	No	Yes	NR	NA	Partially
Mirghani 2010	Yes	Yes	CD	Yes	No	No	Yes	Yes	Partially	No	Partially	No	NA	No
Khasawneh 2020	Yes	Yes	Yes	Yes	No	Yes	Yes	Partially	Yes	No	Yes	No	Yes	Partially
Vellamgot 2022	Yes	Yes	Yes	Yes	No	Yes	Yes	Partially	Yes	No	Yes	No	Yes	Partially
Tavassoli 2010	Yes	Yes	Yes	Yes	No	Yes	Yes	Yes	Yes	No	Yes	No	Yes	No
Surrati 2024	Yes	Yes	NA	Yes	No	Partially	Yes	No	Yes	No	Yes	No	NA	Partially
Seoud 2010	Yes	Yes	Partially	Yes	No	Partially	Yes	No	Yes	No	Yes	No	Yes	Partially
Kouhkan 2021	Yes	Yes	CD	Yes	Yes	Yes	Yes	Yes	Yes	No	Yes	No	No	Yes
Nazarpour 2022	Yes	Yes	NR	Yes	yes	Yes	Yes	Partially	Yes	No	Yes	NR	NR	Yes
Gouda 2025	Yes	Yes	NR	Yes	No	Yes	Yes	Yes	Yes	No	Yes	No	NA	No
Aryavand 2024	Yes	Yes	NR	Yes	Yes	Yes	Yes	Yes	Yes	No	Yes	No	NR	No
Ebrahimi 2021	Yes	Yes	NR	Yes	Yes	Yes	Yes	Yes	Yes	No	Yes	NR	Yes	Yes
Jahromi 2011	Yes	Yes	Yes	Yes	No	Yes	Yes	No	Yes	No	Yes	No	No	No
Khalessi 2014	Yes	Yes	Yes	Yes	No	Yes	Yes	No	Yes	No	Yes	No	Yes	No
Khanghah 2020	Yes	Yes	NR	Yes	No	Yes	Yes	Yes	Yes	No	Yes	Yes	Yes	No
MoghaddamBanaem 2012	Yes	Yes	NR	Yes	No	Yes	Yes	Yes	Yes	No	Yes	NR	Yes	Yes
Najjarzadeha 2022	Yes	Yes	Yes	Yes	Yes	Yes	Yes	Yes	Yes	No	Yes	CD	Yes	Yes
Panahi 2025	Yes	Yes	No	Yes	No	Yes	Yes	Yes	Yes	No	Yes	No	Not	No
ParaparambilVellamgot 2025	Yes	Yes	Yes	Yes	No	Yes	Yes	yes	Yes	No	Yes	CD	Yes	Yes
Rad 2022	Yes	Yes	Yes	Yes	No	Yes	Yes	Yes	Yes	No	Yes	CD	Yes	Yes

Supplementary Table S5. Critical appraisal of case-control studies

Author, year	1. Was the research question or objective in this paper clearly stated and appropriate?	2. Was the study population clearly specified and defined?	3. Was the study population clearly specified and defined?	4. Were controls selected or recruited from the same or similar population that gave rise to the cases (including the same timeframe)?	5. Were the definitions, inclusion and exclusion criteria, algorithms or processes used to identify or select cases and controls valid, reliable, and implemented consistently across all study participants?	6. Were the cases clearly defined and differentiated from controls?	7. If less than 100 percent of eligible cases and/or controls were selected for the study, were the cases and/or controls randomly selected from those eligible?	8. Was there use of concurrent controls?	9. Were the investigators able to confirm that the exposure/risk occurred prior to the development of the condition or event that defined Yesa participant as a case?	10. Were the measures of exposure/risk clearly defined, valid, reliable, and implemented consistently (including the same time period) across all study participants?	11. Were the assessors of exposure/risk blinded to the case or control status of participants?	12. Were key potential confounding variables measured and adjusted statistically in the analyses? If matching was used, did the investigators account for matching during study analysis?
Abdelraheim 2019	Yes	Yes	No	Yes	Partially	Yes	Yes	Yes	Partially	Yes	No	Yes
Alavi 2021	Yes	Yes	Yes	Yes	Yes	Yes	Yes	Yes	Yes	Yes	No	Yes/matching
Al-Kadri 2013	Yes	Yes	No	Yes	Yes	Yes	NA	Yes	Yes	Yes	No	Yes/matching

Alhasoon 2024	Yes	Yes	No	No	Yes	Yes	No	Yes	Yes	Yes	No	Yes/ No matching
Alijahan 2014	Yes	Yes	No	Yes	Yes	Yes	No	Yes	Yes	Yes	No	Partially/Matching done
Ali 2024	Yes	Yes	No	Yes	Yes	Yes	No	Yes	Yes	Yes	No	No
Ali 2017	Yes	Yes	No	Yes	Yes	Yes	No	Yes	Yes	Yes	No	Partially/No matching
AlJama 2012	Yes	Yes	No	Yes	Yes	Yes	No	Yes	Yes	Yes	No	Yes/ No matching
Almaghrabi 2022	Yes	Yes	No	NA	Yes	Yes	NA	NA	Yes	Yes	No	Partially/Matching not applicable
Nasab 2021	Yes	Yes	No	Yes	Yes	Yes	Yes	Yes	Yes	Yes	No	Yes/Matching
ElTaher 2004	Yes	Yes	No	Yes	Partially	Yes	No	Yes	Partially	Partially	No	No
Darine 2021	Yes	Partially	No	Yes	Partially	No	NA	Yes	No	Partially	No	No
Sharami 2021	Yes	Yes	No	Yes	Partially	No	NA	Yes	No	Yes	No	No
Wahabi 2024	Yes	Yes	Yes	Yes	Yes	Yes	NA	Yes	Partially yes	Partially	No	Yes
Khezri 2025	Yes	Yes	No	Yes	Yes	Yes	Yes	Yes	Yes	Yes	No	Yes
Shahgeibi 2009	Yes	Partially	No	Yes	Partially	Yes	NA	Yes	Yes	Partially	No	No
Ziaei 2006	Yes	Yes	Yes	Yes	Yes	Yes	Partially	Yes	Yes	Yes	No	Partially
Tahmasebifard 2025	Yes	Yes	Yes	Partially	Yes	CD	Yes	Yes	Partially	No	Yes	Yes
Yazdani 2015	Yes	Yes	Yes	Yes	Yes	Yes	CD	Yes	Yes	Yes	No	Yes
Gomaa 2021	Yes	Yes	No	Yes	Yes	Yes	Yes	Yes	Yes	Yes	No	Partially/No matching
NamavarJahromi 2008	Yes	Yes	No	Yes	Yes	Yes	Yes	Yes	Partially	Yes	No	No
Borna 2010	Yes	Yes	No	Yes	Yes	Yes	CD	Yes	Yes	Yes	No	Yes
Derakhshi 2014	Yes	Yes	Yes	Yes	Yes	Yes	No	Yes	Yes	Yes	No	Yes
Azizieh 2015	Yes	Yes	No	Yes	Yes	Yes	No	Yes	No	Yes	No	No
Mohammed 2022	Yes	Yes	No	Yes	Yes	Yes	No	Yes	Partially	Yes	No	Yes
Khezri 2025	Yes	Yes	No	Yes	Yes	Yes	Yes	Yes	Yes	Yes	No	Yes
Al-Wassia 2017	Yes	Yes	No	Yes	Yes	Yes	Yes	Yes	Yes	Yes	No	Yes
DahmanHAB 2020	Yes	Yes	No	Yes	Partially	Yes	CD	Yes	Yes	Yes	No	Yes
Hamta 2017	Yes	Yes	No	Yes	Yes	Yes	Yes	Yes	Yes	Yes	No	Yes
Hadavi 2011	Yes	Yes	No	Yes	Yes	Yes	Partially	Yes	Partially	Yes	Not mentioned	Partially
Vaezi 2017	Yes	Yes	No	Yes	Partially	Yes	Yes	Yes	Yes	Partially	No	Partially
Taghavi 2009	Yes	Yes	Yes	Yes	Yes	Yes	Partially	Yes	Yes	Yes	No	Partially
Saleh-Gargari 2009	Yes	Yes	No	Yes	Yes	Yes	NR	Yes	Partially	Yes	No	Partially
Salama 2023	Yes	Yes	No	Yes	Yes	Yes	NR	Yes	Partially	Yes	No	Yes
Rejali 2017	Yes	Yes	Yes	Yes	Yes	Yes	No	Yes	No	Yes	No	Partially
HaghshenasMojaveri 2021	Yes	Yes	No	Yes	Yes	Yes	NR	Yes	Yes	Partially	NR	No
Ibrahim 2015	Yes	Yes	Yes	Yes	Yes	Yes	CD	Yes	Partially	Yes	CD	No
Jafarpour 2025	Yes	Yes	Yes	Yes	Yes	Yes	No	Yes	Yes	Yes	No	Yes
Nair 2024	Yes	No	Yes	Yes	Yes	Yes	Yes	Yes	Yes	No	No	Yes
Najati 2009	Yes	Yes	No	Yes	Yes	Yes	CD	Yes	Yes	Yes	CD	No

Supplementary Table S6. Critical appraisal of randomised-control trials

Author, year	1. Was the study described as randomized, a	2. Was the method of randomization adequate (i.e., use of	3. Was the treatment allocation concealed (so that	4. Were study participants and providers blinded to	5. Were the people assessing the outcomes blinded to	6. Were the groups similar at baseline on important characterist	7. Was the overall drop-out rate from the	8. Was the differential drop-out rate (betwee	9. Was there high adherence to the interventi	10. Were other interventions avoided or similar in	11. Were outcomes assessed using valid and reliable	12. Did the authors report that the sample	13. Were outcomes reported or subgroups	14. Were all randomized participants

	randomized trial, a randomized clinical trial, or an RCT?	randomly generated assignment)?	assignments could not be predicted)?	treatment group assignment?	the participants' group assignments?	ics that could affect outcomes (e.g., demographics, risk factors, co-morbid conditions)?	study at endpoint 20% or lower of the number allocated to treatment?	n treatment groups) at endpoint 15 percentage points or lower?	on protocols for each treatment group?	the groups (e.g., similar background treatments)?	measures, implemented consistently across all study participants?	size was sufficiently large to be able to detect a difference in the main outcome between groups with at least 80% pow	analyzed prespecified (i.e., identified before analyses were conducted)?	analyzed in the group to which they were originally assigned, i.e., did they use an intention-to-treat analysis?
Abdulhussain 2022	Yes	No	No	No	No	Yes	Yes	Yes	Yes	Yes	Partially	No	Yes	Yes
Maher 2013	Yes	Yes	No	No	No	Yes	Yes	Yes	Yes	Yes	Yes	No	Yes	Yes
Zamani 2013	Yes	Yes	CD	Yes	CD	Yes	Yes	Yes	Yes	NR	Yes	No	CD	NR
ZahiriSorouri 2016	Yes	Yes	CD	No	Yes	Yes	Yes	Yes	Yes	Yes	yes	Yes	Yes	Yes
Ebadi 2025	Yes	No	NR	No	NR	Yes	Yes	NR	NR	Yes	Yes	No	Yes	No
Ghomian 2013	Yes	CD	CD	Yes	CD	Yes	Yes	Yes	Yes	Yes	Yes	No	Yes	Yes
Kashanian 2018	Yes	CD	NR	No	No	Yes	Yes	Yes	Yes	Yes	Yes	No	CD	No
Vanda 2024	Yes	Yes	Yes	Yes	Yes	Yes	Yes	Yes	Yes	Yes	Yes	Yes	Yes	Yes
Tara 2010	Yes	Yes	CD	Yes	CD	Yes	No	Yes	Partially	Yes	Yes	No	CD	No
Shahnazi 2017	Yes	Yes	Yes	Yes	Yes	Yes	Yes	Yes	Partially	Yes	Yes	Yes	Yes	Yes
Ouladsahebmadarek 2011	Yes	Partially	CD	Yes	Yes	Yes	Yes	Yes	Yes	Yes	Yes	Partially	Yes	No
Aminisani 2009	Yes	CD	Yes	Yes	Yes	Yes	Yes	Yes	Yes	Yes	Yes	Yes	Yes	No
Khademolkhamseh 2022	Yes	Yes	Yes	Yes	Yes	Yes	Yes	Yes	Yes	Yes	Yes	Yes	Yes	No
Mirzamoradi 2014	Yes	Yes	Yes	Yes	Yes	Yes	Yes	Yes	Yes	Yes	Yes	Yes	Yes	Yes
Nabhan 2014	Yes	Yes	Yes	Yes	Yes	Yes	Yes	Yes	Yes	Yes	Yes	Yes	Yes	Yes

Supplementary Table S7. Thematic coding of determinants and outcomes

Thematic category	Original variables and terms reported in included studies
**Determinants**	
Abuse	Emotional abuse; Domestic violence during pregnancy; Total intimate partner violence; Physical intimate partner violence; Emotional intimate partner violence; Sexual intimate partner violence; Physical abuse; Sexual abuse; Abuse during pregnancy; Physical abuse; Domestic violence.
Lifestyle factors	Irregular tooth brushing; Lifting heavy objects; Physical exercise
Supplementation, nutrition	Vitamin C supplementation; Vitamin D status; Zinc supplementation; Nutritional status; Copper supplementation; Magnesium sulfate supplementation; Iron supplementation; Zinc and copper level in serum;

	Zinc supplementation during second and early third trimester; Selenium supplementation; Lactofem probiotic supplementation in women with cervical cerclage;
Antenatal care	Antenatal care visits
Maternal age	Maternal age; Maternal age over 40; Maternal age over 35
Obstetric history	Grand multiparas; history of abortion; history of PROM; history of PPROM; previous PROM; previous CS; previous history of macrosomia; parity
Chronic medical conditions	Diabetes mellitus; BMI; Chronic disease; Maternal addiction; Obesity class; Periodontal disease; Medical history; Depression; Family history of diabetes; metabolic syndrome; Pre pregnancy BMI; Overweight/obese
Pregnancy complications	Gestational diabetes mellitus; Placental abruption; Preeclampsia; Twin pregnancy; Leaking; Vaginal bleeding during pregnancy; Placenta type; Multiple pregnancies; Cervical incompetence; Polyhydramnios; Oligohydramnios; Spontaneous uterine contractions; Myometrial thickness; Isolated maternal hypothyroxinemia; Gestational weight gain; Hypertensive disorders of pregnancy
Infections	GBS; Current infection; Type of microorganism; Bacterial vaginosis; Trichomonas vaginalis; UTI in pregnancy; Genital tract infection; Infection; Antenatal infection; Serious bacterial infection; Hbsag carrier status
Biochemical profile	Eotaxin -2 concentration; Haemoglobin; WBC; Procalcitonin; ESR; CRP level; Cord blood lead concentration; Platelet; Prolactin concentration; Hematocrit concentration; Blood lead level; Maternal serum CRP; IL-8; Platelet/lymphocyte ratio
Fetal related	Mal-presentation; Malposition; Intrauterine fatal death; Intrauterine growth retardation
Diagnostic test	PAMG-1 test, Ferning test, Nitrazine test; Concentration of fetal fraction; Vaginal ph; Level of tumor necrosis factor TNF; Vaginal discharge concentrations of Beta-human chorionic gonadotropin; MPV; assessment tool (ELISA, ACON, DIMA, CORTEZ); Cervical length; AFI index; Cervical angle; Positive quadruple test; Free _HCG levels
Treatment	Cervical cerclage; Equisetum arvense cream; Vaginal vs. intramuscular progesterone for treatment of recurrent ptb
**Outcomes**	
Gestational outcomes	Preterm birth; Birth weight; Low birth weight; Prematurity; Preterm delivery; Prematurity; Neonatal birth status; Neonatal weight; Gestational age at time of delivery; Small for GA in asthmatic mothers.
Delivery related	Cesarean section; CS for reassuring fetal tests; Induction outcome; Cesarean section in asthmatic women; Mode of delivery
Fetal/neonatal survival indicators	Resuscitation and its types; Neonatal death; Umbilical cord compression; NICU admission; Non-survival among extremely low-birth-weight infants; APGAR score; Fetal tachycardia; Stillbirth; NICU following CS; Apgar score <7 at 5 minutes; Admission to NICU; Intrapartum fetal death; Mechanical ventilation; Surfactant therapy; Corticosteroid use; Fetal distress; APGAR score < 7; Need to resuscitation; Neonate mortality; Apgar score minute 1; Apgar score minute 5; Hospital stay; FHR deceleration; Neonatal intubation; Surfactant; Death; 1st minute APGAR; 5 min APGAR; Umbilical cord status; Perinatal death
Maternal outcomes	Antepartum hemorrhage; Oligohydramnios; Postpartum hemorrhage; Manual removal of the placenta; Amniotic fluid volume; Meconium aspiration; Bleeding; Vaginal bleeding; Placental abruption; Uterine atony; Placental retention; VB + FHR deceleration; Amniotic fluid level; Blood transfusion; Prolonged hospitalization

Maternal infections	Chorioamnionitis; Endometritis; Fever; intra-abdominal infection; Intrapartum pyrexia; Leukocytosis; Maternal infection; Peak intrapartum temperature; Postpartum pyrexia; Surgical site infection; White blood cells count; Wound infection following lower segment CS
Neonatal outcomes	Acute respiratory distress syndrome; Asphyxia; Bronchiolitis; Bronchopneumonia; Cerebral hemorrhage; Clitoromegaly; CNS hemorrhage; Congenital malformation; Conjunctivitis; Death; Death due to pneumatorax; Esophageal atresia; Fetal injury; Head circumference; Hyperbilirubinemia; Inguinal hernia; Intestinal obstruction; IVH; Laryngomalacia; Metabolic acidosis; NEC; Necessity for ventilation; Necrotizing enterocolitis; Neonatal birth injuries; Omphalitis; Osteopenia; Pulmonary hemorrhage; Pulmonary hypoplasia; RDS; Retinopathy of prematurity; Tracheoesophageal fistula; Umbilical hernia
Neonatal infections	Candida colonization in preterm newborns; Clinical sepsis; E.coli infection; Early neonatal sepsis; Sepsis; Late onset GBS infection
**Themes not related to determinants and outcomes**	
Latency	Duration of PROM; PROM longer than 18 hours; Latency period; prolonged ROM; Latency.
Management	Caesarean delivery; Labour pain; Labor induction in patients with PROM; mode of delivery; Antibiotics prophylaxis; Duration of labour; Cesarean section.

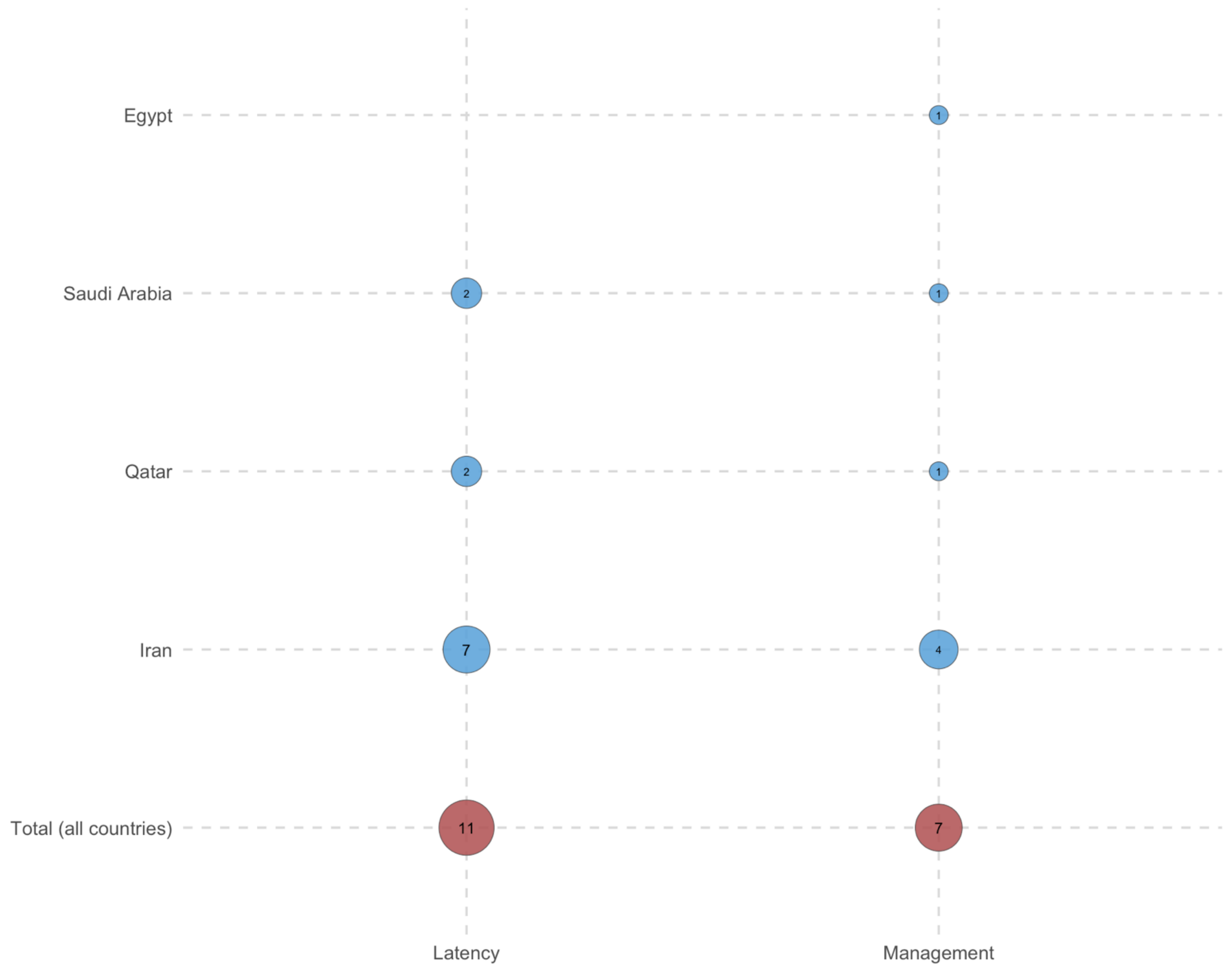

Supplementary Figure S1. Number of statuies on disease course management of PROM in a specific country in the Middle East and Africa.
